# RND efflux pump inhibitor-like natural products as antibiotic adjuvants against multidrug-resistant gram-negative bacteria: a graded-evidence review

**DOI:** 10.3389/fmicb.2026.1915579

**Published:** 2026-09-22

**Authors:** Danxia Kong, Baoyan Liu, Xingchen Ding, Yong Wang

**Affiliations:** 1 Department of Occupational Pulmonology, Shandong First Medical University Affiliated Occupational Disease Hospital (Shandong Province Hospital of Occupational Diseases), Shandong Academy of Occupational Health and Occupational Medicine, Jinan, Shandong, China; 2 Department of Radiation Oncology, Shandong Cancer Hospital and Institute, Shandong First Medical University and Shandong Academy of Medical Sciences, Jinan, Shandong, China

**Keywords:** AcrB, antibiotic adjuvant, efflux pump inhibitor-like candidate, evidence grading, multidrug resistance, natural products, RND efflux pump, structure-feature relationships

## Abstract

Multidrug-resistant (MDR) Gram-negative bacteria—particularly carbapenem-resistant Enterobacterales, *Pseudomonas aeruginosa* and *Acinetobacter baumannii*—remain among the hardest infections to treat, and Resistance-Nodulation-Division (RND) efflux pumps are central to that resistance, making them a recurring target for antibiotic-adjuvant strategies. Natural products offer chemically diverse starting points for efflux-modulating adjuvants, but a fall in MIC is hard to attribute to pump inhibition: membrane permeabilisation, proton-motive-force (PMF) dissipation, efflux-gene downregulation, intrinsic antibacterial activity and general stress responses all give the same readout. We review natural product-derived and semisynthetic RND efflux pump inhibitor-like (EPI-like) candidates in Gram-negative bacteria, organising the evidence by target pump, potentiated antibiotic, proposed mechanism, strength of supporting data and translational feasibility. In place of chemical structures, candidates are read through mechanism-relevant structure-feature associations and the extent of pump-specific validation. Throughout, we separate three routinely conflated claims—antibiotic synergy, functional efflux modulation and direct target binding—and grade them with a pragmatic five-level scheme aimed at curbing over-reading of MIC-based synergy. One pattern dominates: synergy is reported widely, yet candidates reaching pump-associated validation are scarce, and higher-grade pump-dependent evidence remains concentrated on *Escherichia coli* AcrB. Camalexin is the clearest AcrB-dependent phenotype, but only provisionally—a Level 4a grade resting on a single unreplicated study—while plumbagin and nordihydroguaiaretic acid also reach provisional Level 4a because their potentiation and efflux phenotypes were AcrB-dependent and were not explained by outer-membrane permeabilisation. Direct target engagement remains unproven; the rare Level 4 cases beyond AcrB rely on a MexXY-OprM chemical probe or on expression change alone. Comparable pump-specific support for MexB, MexY and AdeB is largely absent. The proposed roadmap should therefore be viewed as an AcrB-anchored hypothesis that requires independent validation across these clinically important pumps. We propose a mechanism-informed structure-feature model and a staged validation roadmap coupling pump-specific strains, membrane and PMF controls, direct target engagement, feature-guided optimisation, cytotoxicity/PK assessment and *in vivo* infection models.

## 1 Introduction

Antimicrobial resistance (AMR) has become one of the leading causes of death worldwide. The Global Burden of Disease analysis linked roughly 4.95 million deaths in 2019 to infections involving bacterial AMR and attributed about 1.27 million directly to resistance; modelling of 1990–2021 trends puts directly attributable deaths on course to approach 1.91 million a year by 2050 (Murray et al., 2022; Naghavi et al., 2024). The pathogens behind this toll are overwhelmingly Gram-negative. Carbapenem-resistant *Acinetobacter baumannii* and carbapenem- or third-generation cephalosporin-resistant Enterobacterales still sit in the World Health Organization critical-priority tier, and carbapenem-resistant *Pseudomonas aeruginosa*, though downgraded from critical to high in the 2024 revision, remains a pressing target (Tacconelli et al., 2018; Sati et al., 2025). In these organisms resistance rarely comes from a single source. The outer-membrane barrier, beta-lactamases, target-site mutations, active efflux, biofilm formation and regulatory rewiring tend to act together, and even the newer beta-lactam/beta-lactamase-inhibitor combinations remain vulnerable to efflux—one reason adjuvant strategies have moved from theoretical appeal to clinical relevance (Wright, 2016; Masi et al., 2017; Laws et al., 2019; Darby et al., 2023; Dulanto Chiang and Dekker, 2024). Of the efflux families, the Resistance-Nodulation-Division (RND) superfamily matters most in Gram-negative bacteria; its members—AcrAB-TolC in *Escherichia coli* and *Klebsiella pneumoniae*, MexAB-OprM and MexXY-OprM in *P. aeruginosa*, and AdeABC in *A. baumannii*—pump multiple antibiotic classes out of the cell and have repeatedly been tied to virulence and biofilm formation (Kvist et al., 2008; Du et al., 2018; Colclough et al., 2020; Nishino et al., 2021; Kavanaugh et al., 2024). Blocking RND-mediated efflux should, in principle, let antibiotics accumulate and restore agents that resistance has compromised. Yet after decades of effort none has reached the clinic, held back by the structural intricacy of these tripartite machines, the difficulty of inhibiting them without membrane toxicity, and the unsolved question of which antibiotic to pair them with and how to dose it (Sharma et al., 2019; Alav et al., 2021; Compagne et al., 2023). The wider framing is by now familiar: AMR is treated as a systemic threat to modern medicine and to global health security (Cheng et al., 2016; Salam et al., 2023). Against that backdrop, adjuvant and resistance-breaking approaches—efflux inhibition, antibiotic hybrids and rational combinations—keep being proposed as ways to prolong the usefulness of drugs already in hand (Spengler et al., 2017; Domalaon et al., 2018; Annunziato, 2019). Synthetic EPI programmes, surveyed across chemical series and the critical-priority pathogens, capture both the attraction of this target class and the development hurdles that remain (Wang et al., 2016; Lamut et al., 2019; Almatar et al., 2021; Cernicchi et al., 2021).

Natural products are an appealing answer to this gap. They span chemically diverse classes that can shift bacterial physiology, antibiotic accumulation and resistance phenotypes, and their scaffold diversity, stereochemical richness and three-dimensional complexity may suit the large, flexible recognition regions of membrane transporters. That same diversity is also the catch. A compound may lower resistance through pump engagement, indirect regulatory effects, membrane perturbation, PMF dissipation or non-specific antibacterial stress, and partly for that reason plant secondary metabolites have been labelled, almost interchangeably, as direct EPIs, antibiotic potentiators, resistance modifiers and semisynthetic starting points—labels that carry different evidential weight (Stavri et al., 2007; Cheesman et al., 2017; Seukep et al., 2020; Seukep et al., 2022). Flavonoids, alkaloids, terpenoids, essential-oil components, coumarins, quinones and phenolic acids are repeatedly reported to behave as EPI-like compounds (Ohene-Agyei et al., 2014; Waditzer and Bucar, 2021; Dias et al., 2022). The more telling endpoint, however, is not synergy but whether the effect can be tied to a specific pump. Few candidates clear that bar; plumbagin and nordihydroguaiaretic acid (NDGA), linked to *E. coli* AcrB-associated phenotypes and hydrophobic-pocket reasoning, are among the rare examples (Ohene-Agyei et al., 2014). This review therefore concentrates on defined natural products and semisynthetic derivatives reported to modulate RND-family pumps or RND-associated phenotypes in Gram-negative bacteria, asking not merely whether they lower an MIC but whether the evidence supports a pump-specific, mechanistically interpretable and translatable EPI-like claim.

The review’s central message is that phenotypic synergy is reported in abundance, whereas genuine Gram-negative RND pump-associated validation is scarce and concentrated mainly on *E. coli* AcrB. Earlier reviews have catalogued natural-product efflux modulators and their reported synergy, but MIC reduction and pump-specific inhibition are often treated as if they were the same endpoint. We keep these claims separate and make their evidential strength explicit. Three features define the review: a five-level hierarchy that distinguishes antibiotic synergy, functional efflux modulation and direct target engagement—and separates pump-dependent phenotypes (Level 4a) from expression change alone (Level 4b); a mechanism-informed structure-feature model based on experimentally interpretable features rather than chemical drawings; and a staged validation roadmap coupling pump-specific strains, membrane and PMF controls, direct target engagement, feature-guided optimisation and *in vivo* testing.

### 1.1 Literature search and review approach

To improve transparency, we conducted this work as a narrative, evidence-graded review rather than a formal systematic review. PubMed/MEDLINE, Web of Science Core Collection and Scopus were searched for English-language, peer-reviewed literature available up to 20 April 2026. Search terms combined natural products and major scaffold classes with efflux pump inhibitor, antibiotic adjuvant, RND, AcrB, MexB, MexY, AdeB, AcrAB-TolC, MexAB-OprM, MexXY-OprM and AdeABC. Reference lists of relevant reviews and primary studies were also screened. Defined natural products or semisynthetic derivatives tested in Gram-negative bacteria with MIC/FICI, accumulation/efflux, pump-expression, pump-strain, binding or *in vivo* data were considered core or near-core evidence. Crude extracts, Gram-positive or non-RND pump studies and docking-only reports were retained only when they helped interpret mechanism or structure-feature hypotheses and were therefore classified as auxiliary rather than pump-specific RND validation.

## 2 Structural and functional basis of RND efflux pumps

### 2.1 Tripartite architecture

RND efflux pumps cross the entire Gram-negative envelope as tripartite complexes: an inner-membrane RND transporter, a periplasmic membrane-fusion/adaptor protein and an outer-membrane channel (Figure 1) (Du et al., 2018; Alav et al., 2021). AcrAB-TolC, MexAB-OprM, MexXY-OprM and AdeABC are the prototypes. Crystallography and cryo-EM have shown how the three parts dock into a continuous conduit running from the periplasm—including the periplasmic face of the inner membrane—to the cell exterior (Murakami et al., 2006; Daury et al., 2016; Shi et al., 2019; Tsutsumi et al., 2019). Broader surveys of the superfamily, together with recent high-resolution work on diverse transporters, have pushed this picture well past the prototypical AcrB system (Athar et al., 2023; Klenotic and Yu, 2024). Because the assembly bridges both membranes, its behaviour cannot be read in isolation from the outer-membrane permeability barrier and porin-mediated influx; the physicochemical rules that govern Gram-negative entry are now weighed alongside efflux whenever drug accumulation is interpreted (Richter et al., 2017; Zgurskaya et al., 2018; Parker et al., 2019; Prajapati et al., 2021).

**Figure 1:**
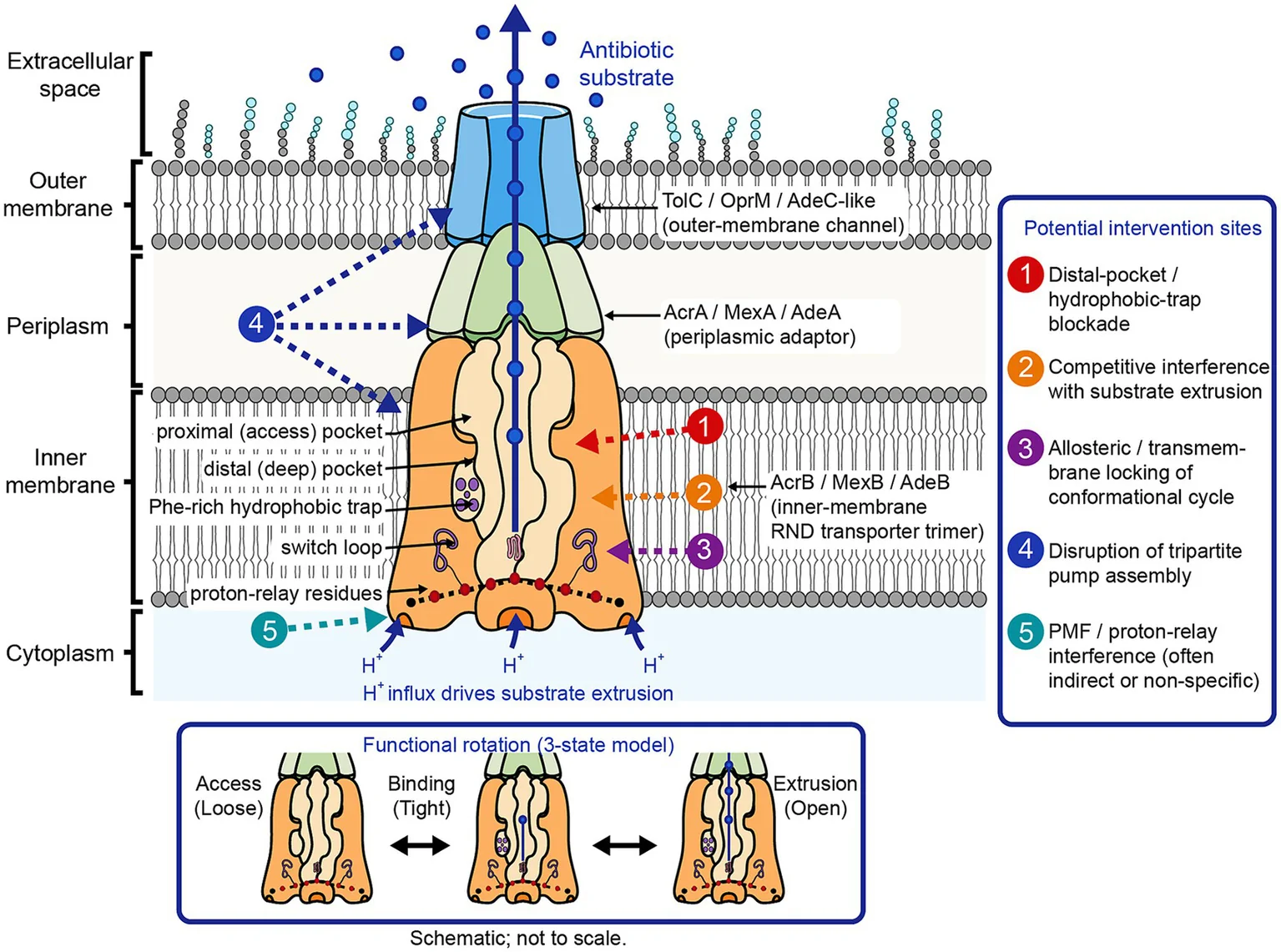
Architecture and potential inhibition sites in resistance-nodulation-division (RND) efflux pumps. The tripartite complex comprises an inner-membrane RND transporter, a periplasmic adaptor and an outer-membrane channel. The transporter trimer cycles through access, binding and extrusion states during functional rotation. Potential intervention points include substrate-pocket blockade, conformational locking, disruption of pump assembly and interference with the proton motive force (PMF).

### 2.2 Functional rotation mechanism

AcrB, MexB and AdeB work as homotrimers, each protomer cycling through access, binding and extrusion states in what is usually called functional rotation (Murakami et al., 2006). Capture, tight binding and extrusion are mechanically coupled to proton flow through conserved transmembrane residues (Eicher et al., 2014; Zwama and Yamaguchi, 2018; Kobylka et al., 2020). An inhibitor could therefore intervene at several points: occluding a substrate pocket, competing with substrate, stalling the conformational cycle, uncoupling proton flow or blocking assembly. Free-energy calculations of stepwise translocation, and allosteric models of the AcrAB-TolC and MexAB-OprM assemblies, add detail on how these transitions track drug movement (Zuo et al., 2015; Wang et al., 2017; Glavier et al., 2020).

### 2.3 Substrate-binding pockets and the hydrophobic trap

AcrB and its homologues carry several substrate-recognition regions—a proximal (access) pocket, a distal (deep) pocket, a switch loop and a phenylalanine-rich hydrophobic trap (Vargiu and Nikaido, 2012; Nakashima et al., 2013). Synthetic inhibitors such as the pyranopyridines and MBX-class compounds bind within this region, and a stronger hydrogen-bond network in the distal pocket raises potency (Vargiu et al., 2014; Sjuts et al., 2016; Aron and Opperman, 2018). Natural products that combine aromatic, hydrophobic and hydrogen-bonding features may engage the same sites, but the claim stays inferential without binding, mutagenesis or structural data. Structural and simulation studies of how carbapenems and other substrates sit in the AcrB pocket—and comparative work on the promiscuous recognition shared by AcrB and AdeB—give a reference frame for judging putative natural-product binding (Atzori et al., 2020; Ornik-Cha et al., 2021).

### 2.4 Proton motive force and energy coupling

RND transport runs on the proton-motive force, with drug export coupled to inward proton movement through conserved titratable residues in the transmembrane domain (Du et al., 2018; Kavanaugh et al., 2024). A compound that collapses the PMF will lower efflux too, but the effect is non-specific and may signal nothing more than membrane toxicity. PMF and membrane-potential controls are, for that reason, indispensable when a natural product’s potentiation is interpreted (Blair and Piddock, 2016; Plé et al., 2022).

### 2.5 Clinically important RND pumps

Gram-negative pathogens draw on overlapping but non-identical sets of RND pumps (Table 1): *E. coli* relies mainly on AcrAB-TolC; *K. pneumoniae* carries AcrAB-TolC alongside OqxAB; *P. aeruginosa* uses MexAB-OprM, MexXY-OprM and MexCD-OprJ; and *A. baumannii* uses AdeABC, AdeIJK and AdeFGH (Colclough et al., 2020; Avakh et al., 2023; Kavanaugh et al., 2024). These pumps differ in substrate range, regulation and conformational plasticity, limiting extrapolation from one organism or transporter to another (Lazarova et al., 2025). Evidence beyond the prototypical *E. coli* AcrB system includes high-resolution structures of *K. pneumoniae* AcrB and functional analysis of OqxB, which contributes to fluoroquinolone and tigecycline resistance (Bharatham et al., 2021; Zhang et al., 2023). Expression is controlled by overlapping regulatory networks: altered activity of regulators such as *marA*, *soxS* and *ramA* maps onto AcrAB-mediated multidrug resistance, and two-component systems add further control points (Ferrari et al., 2013; Tiwari et al., 2017).

**Table 1:** Clinically important RND efflux pumps in representative Gram-negative pathogens.

Species	Major RND pump(s)	Components	Representative reported substrates or affected antibiotic classes	Regulators/clinical relevance
*E. coli*	AcrAB-TolC	AcrB / AcrA / TolC	Beta-lactams, fluoroquinolones, tetracyclines, chloramphenicol	*marA, ramA, soxS*; prototype Gram-negative pump
*K. pneumoniae*	AcrAB-TolC; OqxAB	AcrB/AcrA/TolC; OqxB/OqxA	Fluoroquinolones, tigecycline, olaquindox; selected disinfectant/biocide-associated substrates	*ramR, rarA*; tigecycline non-susceptibility
*P. aeruginosa*	MexAB-OprM; MexXY-OprM; MexCD-OprJ	MexB/MexA/OprM; MexY/MexX/OprM; MexD/MexC/OprJ	Beta-lactams, fluoroquinolones, aminoglycosides, chloramphenicol	*mexR, nalC, nalD, mexZ, nfxB*; intrinsic and acquired resistance
*A. baumannii*	AdeABC; AdeIJK; AdeFGH	AdeB/AdeA/AdeC; AdeJ/AdeI/AdeK; AdeG/AdeF/AdeH	Tigecycline, fluoroquinolones, aminoglycosides	*adeRS* and additional regulators; tigecycline and multidrug resistance

Sources: Kavanaugh et al. (2024), Colclough et al. (2020), Avakh et al. (2023), and Du et al. (2018). RND, resistance-nodulation-division.

## 3 Evidence-grading framework for natural product-derived RND EPI-like candidates

### 3.1 Why evidence grading is necessary

A recurring weakness in the natural product EPI literature is the leap from a fall in MIC, or checkerboard synergy, to RND-specific inhibition. The same potentiation can come from pump-targeted inhibition, outer-membrane permeabilisation, inner-membrane damage, PMF dissipation, intrinsic antibacterial activity, increased uptake, efflux-gene downregulation, biofilm inhibition or shifts in virulence (Compagne et al., 2023; Duffey et al., 2024). Because these routes share a phenotype, claims of different strength can only be compared on common ground if the evidence is graded.

### 3.2 Five-level evidence hierarchy

The hierarchy adds no new assays. It takes controls already standard in efflux work—the dye-accumulation, membrane-integrity and pump-strain controls of Blair and Piddock, and the benchmark-inhibitor approach of Lomovskaya and colleagues—and arranges them into ordinal levels so that claim strength becomes explicit and traceable across compounds (Lomovskaya et al., 2001; Blair and Piddock, 2016). The contribution is organisational, not experimental: qualitative but widely endorsed control recommendations are recast as an explicit scale. We offer the five-level scheme (Table 2; Figure 2) as a working framework, not a validated consensus standard. Level 1 (docking, simulation or target prediction alone) is hypothesis-generating only. Level 2 (MIC reduction, checkerboard/FICI [fractional inhibitory concentration index] synergy or time-kill) marks antibiotic potentiation but says nothing about mechanism. Level 3 (EtBr [ethidium bromide], Nile Red or Hoechst accumulation with efflux kinetics) points to functional efflux modulation but still needs membrane and viability controls. Level 4 supports pump association without proving direct binding and is split because a demonstrated pump-dependent phenotype and a change in pump abundance are not equivalent. Level 4a is a pump-dependent phenotype shown with isogenic knockout, overexpression or complementation strains, whereas Level 4b is a change in pump expression alone (qPCR or protein level) without a genetic dependence test. Level 5 calls for direct target engagement, mutational mapping, structural validation, resistant-mutant analysis or pump-linked *in vivo* efficacy. *In vivo* efficacy qualifies for Level 5 only when the outcome is explicitly linked to the nominated pump or target mechanism; animal efficacy without pump-specific proof remains at the level supported by its underlying mechanistic data. Where evidence straddles two adjacent levels, we retain a hyphenated grade (for example, L2–3) to show the full evidence range, while the lower level governs conservative interpretation. The suffix “auxiliary” flags evidence from Gram-positive or non-RND systems that informs structure-feature reasoning but does not count as Gram-negative RND validation.

**Table 2:** Five-level evidence hierarchy for natural product-derived RND EPI-like activity.

Level	Evidence type	Interpretation	Main limitation/recommended controls
L1	Docking, MD simulation, target prediction only	Hypothesis-generating only	No experimental support; require biological assays
L2	MIC reduction, checkerboard/FICI, time-kill	Possible antibiotic potentiator	Mechanism unknown; false-positive risk includes intrinsic antibacterial activity and growth inhibition; include compound-alone MIC and benchmark controls
L3	EtBr/Nile Red/Hoechst accumulation, efflux kinetics	Functional efflux modulation	Cannot exclude membrane permeabilisation, PMF collapse or cell death; add NPN/PI, membrane-potential, ATP and viability controls
L4a	Pump-dependent phenotype shown with isogenic knockout, overexpression or complementation strains	Strong pump-dependence (the pump is required for the phenotype)	Does not prove direct binding; confirm with target-engagement assays (Level 5)
L4b	Change in pump expression only (qPCR or protein level), without an isogenic pump-dependence test	Regulatory pump-association only; not evidence of transporter inhibition	Expression change neither requires the pump for the phenotype nor proves direct inhibition; false-positive risk includes general stress responses; add isogenic pump strains and functional assays
L5	Direct binding, mutagenesis, resistant-mutant mapping, cryo-EM/X-ray, or *in vivo* validation linked to pump mechanism	Strong target or translational validation	Resource-intensive; reserve for causal claims and candidate advancement; in vivo efficacy counts as Level 5 only when linked to a pump mechanism

Evidence progresses from computational prediction and phenotypic potentiation to functional efflux modulation, pump-associated validation and direct target or translational evidence. Level 4a indicates a pump-dependent phenotype, whereas Level 4b indicates altered pump expression without genetic dependence. MIC reduction or synergy alone does not establish RND-specific inhibition. ATP, adenosine triphosphate; cryo-EM, cryogenic electron microscopy; EPI, efflux pump inhibitor; EtBr, ethidium bromide; FICI, fractional inhibitory concentration index; MD, molecular dynamics; MIC, minimum inhibitory concentration; NPN, N-phenyl-1-naphthylamine; PI, propidium iodide; PMF, proton-motive force; qPCR, quantitative polymerase chain reaction.

**Figure 2:**
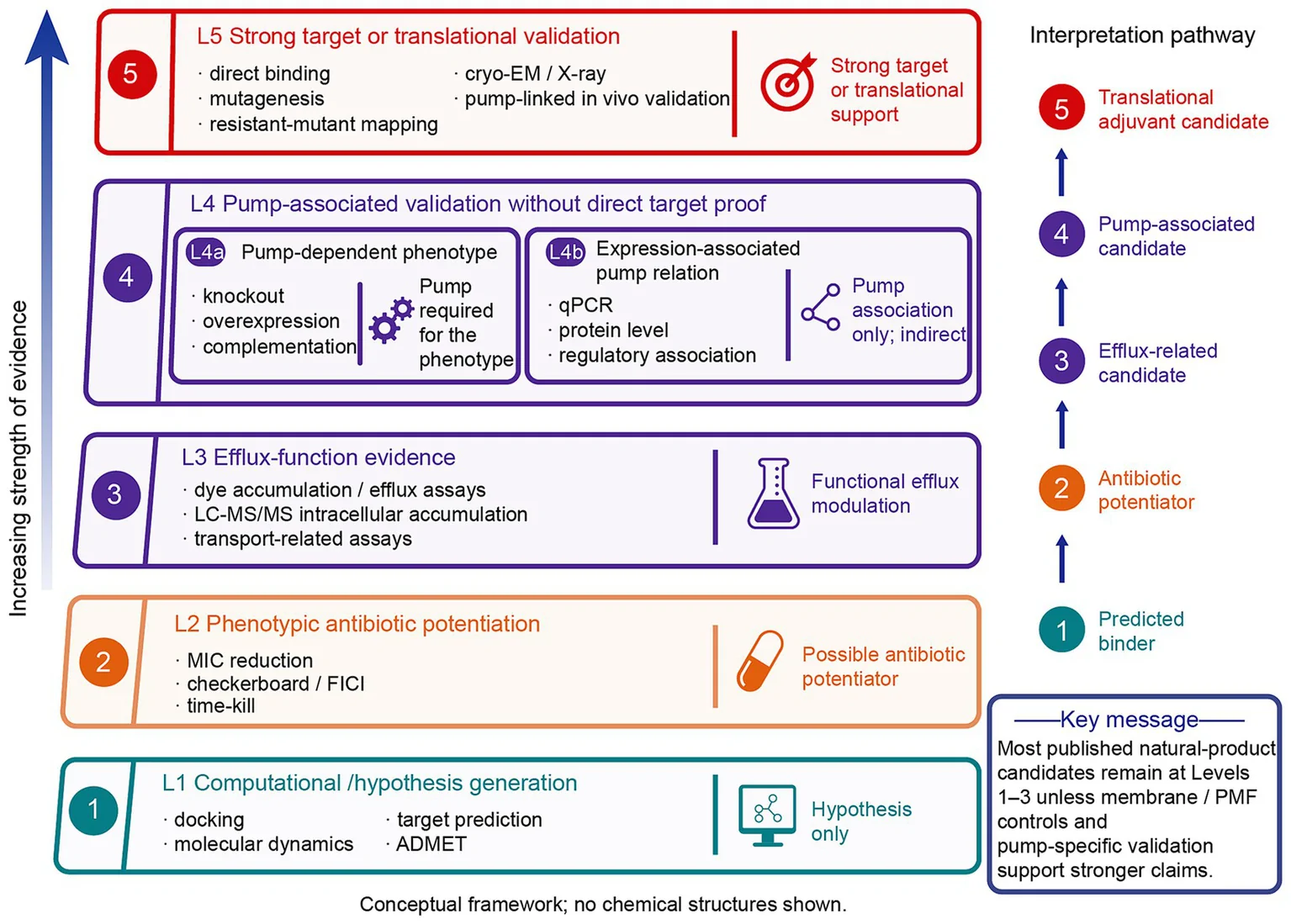
Graded evidence framework for natural product-derived RND EPI-like candidates. The five-level framework separates computational prediction, phenotypic potentiation, functional efflux modulation, pump-associated evidence and strong target or translational validation. The interpretation pathway emphasises progression from hypothesis generation to translational adjuvant candidates and highlights the need for membrane/PMF controls and pump-specific validation to support stronger claims.

### 3.3 Minimum validation standards

A minimal validation package would cover compound-alone and antibiotic-combination MICs with FICI, an accumulation or efflux assay, membrane-permeability and membrane-potential/PMF controls, growth and viability controls, cytotoxicity and haemolysis, at least one pump-deficient or pump-overexpressing strain, at least one clinically relevant MDR isolate, and a benchmark EPI or membrane-active control (Lomovskaya et al., 2001; Opperman et al., 2014; Iyer et al., 2018). Anything stronger than pump association then needs direct binding, mutagenesis, resistant-mutant mapping, structural analysis or animal infection models. Validated accumulation methods— quantitative LC–MS/MS assays and predictive tools for intrabacterial concentration—tighten the package by measuring real changes in intracellular drug rather than inferring them from dye fluorescence (Geddes et al., 2021).

### 3.4 Worked example: applying the framework to camalexin

It helps to follow one compound through the scheme. Camalexin, an indole phytoalexin, raised ethidium accumulation in *E. coli*—on its own a Level 3 result. The same study found no parallel increase in outer-membrane permeability or loss of membrane integrity, and potentiation disappeared in an AcrB-inactive background; docking placed camalexin in an AcrB pocket (Irianti et al., 2025). Because the phenotype depended on functional AcrB rather than merely an expression change, camalexin reaches provisional Level 4a. It stops short of Level 5 because direct binding, residue-level mapping, resistant-mutant analysis and pump-linked *in vivo* efficacy have not been reported. The grade also rests on a single primary study and therefore requires independent replication.

The same logic explains why most flavonoids remain at Level 2–3: synergy and dye-accumulation data may exist, but membrane/PMF controls or isogenic pump strains are usually missing. Every compound in Tables 3, 4 was assigned by this sequence, with the evidential basis recorded in the final columns.

**Table 3:** Core or near-core Gram-negative RND EPI-like candidates.

Compound	Class/model	Pump-associated evidence	Antibiotic(s) potentiated	Evidence level	Main limitation	Next validation
Camalexin	Indole phytoalexin; *E. coli*	Ethidium accumulation; no reported membrane permeabilisation; AcrB-dependent phenotype; docking to AcrB pocket	Multiple AcrB substrates	Provisional L4a	Single-study dependence; no binding, mutagenesis or in vivo data	Replicate in independent strains; direct binding; pocket mutants; mechanism-guided analogue testing; PK/toxicity
Plumbagin / NDGA	Naphthoquinone (plumbagin) / phenolic lignan (NDGA); *E. coli*	AcrB-dependent potentiation in wild-type but not Δ*acrB* cells; Nile Red efflux inhibition; no outer-membrane permeabilisation; docking rationale	AcrB-substrate antibiotics	Provisional L4a	Single-study dependence; direct binding and residue-level target engagement remain unproven; redox liabilities possible	Independent replication; direct binding or competition assay; AcrB pocket mutants; redox-liability and membrane/PMF controls
Di-berberine probes	Alkaloid derivatives; *P. aeruginosa*	MexXY-OprM-associated chemical-probe evidence	Aminoglycosides	L4a (probe evidence)	Probe-like rather than drug-like; therapeutic window unresolved	Therapeutic optimisation; toxicity/PK; in vivo combination testing
(+)-Usnic acid	Lichen dibenzofuran; *A. baumannii*	*adeB* expression-associated tigecycline adjuvant effect	Tigecycline	L4b (expression-associated)	Expression change does not prove direct AdeB inhibition or AdeB-dependent phenotype	AdeB-specific strains; accumulation assays; membrane/PMF controls; direct AdeB assays
Cumin and cinnamon essential oils	Essential-oil components; *Salmonella*	AcrAB/AcrD-associated expression and phenotype; candidate constituents proposed by in silico analysis	Multiple antibiotics	L3 (mixture-level expression/phenotype signal)	Mixture/active-principle ambiguity; membrane and PMF artefacts possible; should not be presented as pump-specific RND inhibition	Define active compounds; isogenic pump strains; NPN/PI/PMF controls
Quercetin derivatives	Flavonoid; Gram-negative models	Antibiotic potentiation with efflux-associated or mixed carbapenemase/efflux phenotypes	Carbapenems and others	L2–3	Pump specificity and membrane/PMF controls often incomplete	Matched analogue testing; pump mutants; LC–MS/MS accumulation; binding assays
Lysergol derivatives	Clavine alkaloid; *E. coli*	Efflux-associated synergy and accumulation; proposed efflux interference	Nalidixic acid; tetracyclines/fluoroquinolones	L2–3	No isogenic pump strains or direct target assays	Isogenic pump strains; direct target/accumulation assays; mechanism-guided analogue testing
Ursolic acid derivatives	Semisynthetic triterpenoid; *E. coli*	Efflux-related potentiation in nalidixic acid−/multidrug-resistant *E. coli*	Nalidixic acid; tetracycline	L2–3	Pump specificity unresolved; matched SAR and toxicity data needed	Pump-specific validation; matched analogue testing; cytotoxicity/PK
EGCG (with curcumin evidence)	Polyphenol; *P. aeruginosa* and *A. baumannii*	MexAB-OprM-associated EGCG phenotype in *P. aeruginosa*; separate EGCG–curcumin synergy in *A. baumannii*	Chloramphenicol, tetracyclines and other agents; curcumin combination	L2	EGCG is antibacterial and may also behave as a pump substrate; direct inhibition is not established; membrane and multi-target effects remain possible	Pump-deficient/overexpressing strains; membrane/PMF controls; accumulation assays
Chlorogenic acid	Phenolic acid; *K. pneumoniae*	Levofloxacin potentiation, antibiofilm activity and mouse infection signal; no efflux mechanism established	Levofloxacin	L2	In vivo combination signal is relevant, but pump specificity and efflux contribution were not tested	Pump-specific strains; accumulation assays; membrane/PMF controls

Candidates are summarised by chemical class, organism or pump, potentiated antibiotic, key evidence, assigned evidence grade, principal limitation and next validation step. Level 4 indicates pump association rather than proven direct inhibition. For mixed or hyphenated grades, the lower level governs conservative interpretation. EGCG, epigallocatechin gallate; EPI, efflux pump inhibitor; LC–MS/MS, liquid chromatography–tandem mass spectrometry; NDGA, nordihydroguaiaretic acid; PK, pharmacokinetics; PMF, proton-motive force; RND, resistance-nodulation-division.

**Table 4:** Auxiliary evidence not considered pump-specific Gram-negative RND validation.

Compound	Class / model	Pump-associated evidence	Antibiotic(s) potentiated	Evidence level	Main limitation	Next validation
Carvacrol / thymol	Phenolic monoterpenes; mostly NorA/*Salmonella*-related evidence	Efflux-like potentiation and expression effects; frequent membrane activity	Fluoroquinolones and others	L2–3 (auxiliary)	Membrane perturbation and non-RND evidence dominate	Use only as optimisation/formulation candidates until Gram-negative RND controls are complete
Nerolidol / farnesol / bisabolol	Sesquiterpenes; mainly Gram-positive models	NorA/non-RND or efflux-like phenotypes	Norfloxacin and related agents	L2–3 (auxiliary)	Useful formulation/mechanism evidence but not direct RND proof	Test defined compounds in AcrB/MexB/AdeB systems with membrane/PMF controls
Amino-chalcones	Semisynthetic flavonoids; mostly NorA/MepA models	Efflux-associated activity in non-RND systems	Fluoroquinolones	L2–3 (auxiliary)	Mechanism-relevant but should not be cited as Gram-negative RND evidence	Re-test in Gram-negative pump-mutant panels
Berberine / reserpine comparators	Alkaloid-like historical comparators	Substrate/inhibitor behaviour varies by pump and species	Multiple agents	Auxiliary	Strongest evidence often non-RND or Gram-positive	Use as benchmarks only, not as validated pump-specific candidates
Crude plant extracts	Defined-compound status unresolved; extract-level screening evidence	Potentiation or dye accumulation without defined active compound(s)	Various agents	Auxiliary / screening only	Batch variability, composition uncertainty and membrane effects prevent compound-level RND claims	Fractionate and identify active principles; test defined compounds with pump-specific strains and membrane/PMF controls
Docking-only compounds	Predicted AcrB/MexB/AdeB pocket binding without biological validation	Not established experimentally	Not established	L1 (hypothesis-generating)	No experimental support; cannot establish efflux modulation or pump dependence	Add MIC/FICI, accumulation/efflux, membrane/PMF and target-engagement assays
PMF disruptors / membrane-active natural products	Terpenoids, phenolics, quinones and other membrane-active scaffolds	Potentiation may reflect PMF collapse, permeability change, intrinsic antibacterial activity or cytotoxicity	Multiple antibiotics	L2–3 (auxiliary unless controls are negative)	Mechanism frequently non-specific; pump association cannot be inferred without negative membrane/PMF controls	Include NPN/PI, membrane potential, ATP, viability and pump-mutant controls before EPI claims

These studies mainly involve Gram-positive or non-RND pumps, crude extracts, mixed mechanisms, membrane-active compounds or docking-only evidence. They inform hypothesis generation and assay design but are not counted as direct Gram-negative RND evidence. ATP, adenosine triphosphate; EPI, efflux pump inhibitor; FICI, fractional inhibitory concentration index; MIC, minimum inhibitory concentration; NPN, N-phenyl-1-naphthylamine; PI, propidium iodide; PMF, proton-motive force; RND, resistance-nodulation-division.

### 3.5 How candidates were classified as core or auxiliary

We treat a compound as a core or near-core RND EPI-like candidate only when it is a defined natural product or derivative, tested in Gram-negative bacteria, tied to an RND pump or RND-associated phenotype, and backed by at least MIC/FICI or efflux-assay data—ideally with pump-strain, expression, binding or *in vivo* evidence as well. Compounds resting only on generic antibacterial MICs, Gram-positive NorA data, MFS/MATE/ABC-pump data, crude extracts or docking are filed as auxiliary or hypothesis-generating, and non-RND data are kept only as comparative, mechanism-generating or structure-feature background. Two authors assigned levels independently from the criteria in Table 2 and settled disagreements by discussion. No formal inter-rater statistic such as Cohen’s kappa was calculated; consequently, the grading should be regarded as transparent and auditable but still qualitative, rather than as a quantitatively validated reproducibility measure. Each candidate was judged against the same fixed criteria in Table 2, and the evidential basis for every grade is reported in the final two columns of Tables 3, 4. Future applications should use a per-criterion checklist and report an agreement statistic. Where direct target-engagement data are missing, we prefer the terms EPI-like, efflux-modulating or pump-associated candidate to any claim of direct RND inhibition.

## 4 Integrated class-by-class evidence, mechanisms and structure-feature relationships

This section integrates, for each chemical class, the reported phenotype, proposed mechanism, relevant structure-feature hypotheses and validation gaps. Candidates fall into three tiers: core or near-core Gram-negative RND candidates with pump-associated evidence; intermediate candidates with Gram-negative efflux or synergy data but incomplete pump specificity; and auxiliary or non-RND evidence that is useful for mechanistic reasoning but not pump-specific proof. Table 3 and the standalone distribution bar chart in Figure 3 make the imbalance visible. Among the ten core or near-core candidate entries, the assigned evidence-grade categories are L2 (*n* = 2), L2–3 (*n* = 3), L3 (*n* = 1), L4a (*n* = 3), L4b (*n* = 1) and L5 (*n* = 0). Thus, four entries reach a Level 4 grade, and none reaches Level 5.

**Figure 3:**
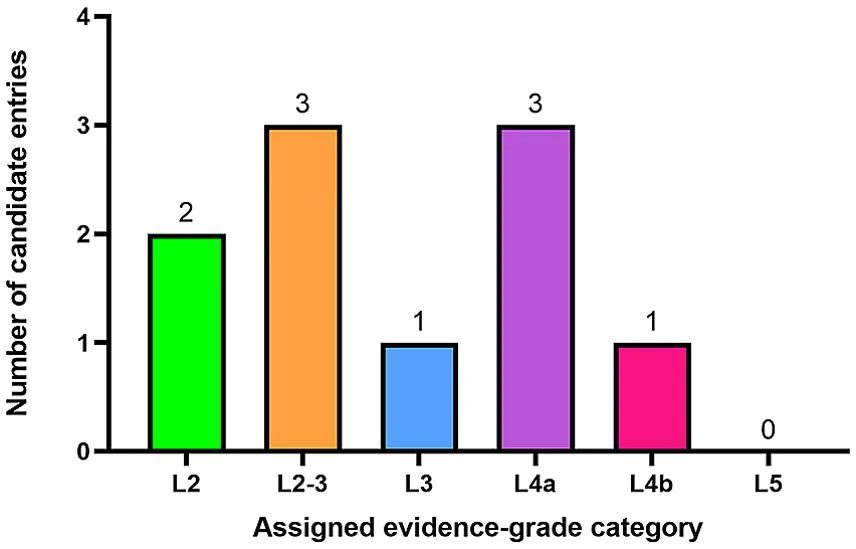
Distribution of core or near-core candidates by assigned evidence-grade category. The ten core or near-core candidate entries classified in Table 3 are distributed as follows: L2 (*n* = 2), L2–3 (*n* = 3), L3 (*n* = 1), L4a (*n* = 3), L4b (*n* = 1) and L5 (*n* = 0). The chart makes explicit that lower-level synergy or efflux-modulation evidence greatly outnumbers pump-associated validation, and that no candidate currently reaches Level 5.

### 4.1 Flavonoids and polyphenols

Flavonoids and polyphenols are among the most frequently nominated EPI-like compounds, including quercetin, EGCG, baicalein, chrysin, naringenin, catechin, myricetin, resveratrol, gallotannins and chalcones (Górniak et al., 2019; Waditzer and Bucar, 2021; Shamsudin et al., 2022). In Gram-negative organisms, however, the evidence rarely rises above synergy or functional accumulation. Quercetin derivatives have been associated with antibiotic potentiation, mixed carbapenemase/efflux modulation and dye-accumulation or docking-supported activity (Pal and Tripathi, 2020; Lee et al., 2024). A defined gallotannin increased ethidium accumulation and reduced antibiotic MICs in multidrug-resistant uropathogenic *E. coli*, but the absence of an isogenic RND-pump dependence test keeps the result at functional, not pump-specific, evidence (Bag and Chattopadhyay, 2014). EGCG/curcumin combinations are active against MDR *A. baumannii*, and EGCG has been linked to MexAB-OprM-associated activity in *P. aeruginosa*, but neither case isolates a pump-specific mechanism (Betts and Wareham, 2014; Kanagaratnam et al., 2017). Mechanistic findings from Gram-positive NorA or MepA—including prenylated isoflavonoid studies—cannot be transferred directly to Gram-negative RND systems: NorA/MepA are single inner-membrane transporters in organisms without an outer membrane, whereas AcrAB-TolC, MexAB-OprM and AdeABC are tripartite trans-envelope assemblies with different access constraints, substrate pathways and pump-dependence requirements (Irianti et al., 2023). Such data therefore inform only auxiliary structure-feature hypotheses. Planarity, the C2 = C3/4-keto pattern, hydroxylation versus methoxylation, glycosylation, lipophilicity and polar surface area are plausible optimisation variables, but matched series must be tested with membrane/PMF controls and pump-mutant panels before any RND-specific conclusion is drawn.

### 4.2 Alkaloids

Alkaloids combine cationic or basic nitrogen centres with aromatic and hydrophobic surfaces that may interact with transporter pockets, but the same features can also promote mammalian-cell toxicity, non-selective accumulation or substrate-like behaviour. Lysergol and semisynthetic derivatives potentiated nalidixic acid against resistant *E. coli*, but the available synergy and accumulation evidence lacks isogenic pump strains or direct target assays and therefore remains Level 2–3 (Maurya et al., 2013). Berberine illustrates why behaviour must be described in a pump- and organism-specific manner. At low concentrations in *K. pneumoniae*, berberine upregulated the non-RND KmrA pump and produced a hormetic survival phenotype; in an *E. coli* MdfA model, it was reported to inhibit the MFS transporter and increase intracellular ciprofloxacin (Li et al., 2021; Li and Ge, 2023). Solasodine activity against *S. aureus* NorA is likewise auxiliary rather than evidence for Gram-negative RND inhibition (Da Silva et al., 2022). Di-berberine conjugates provide the more relevant RND-associated evidence as chemical probes of *P. aeruginosa* MexXY-OprM, but their Level 4a designation is probe-level rather than proof of a drug-like lead (Kavanaugh et al., 2023). Future analogue series should vary charge, linker length and hydrophobicity while measuring pump-mutant selectivity, intracellular accumulation, cytotoxicity, haemolysis, solubility, PK and direct target engagement.

### 4.3 Terpenoids and essential-oil components

Terpenoids and essential-oil constituents—carvacrol, thymol, eugenol, geraniol, limonene, linalool, nerolidol, farnesol and alpha-bisabolol among them—often potentiate antibiotics but also perturb membranes, so lipophilicity and membrane affinity can imitate EPI activity (Dias et al., 2022). NorA-centred studies of carvacrol, thymol and sesquiterpenes remain auxiliary rather than core RND evidence (Dos Santos Barbosa et al., 2021; Oliveira-Tintino et al., 2023; Santana et al., 2023). Eugenol can also kill carbapenem-resistant *K. pneumoniae* through reactive-oxygen-species mechanisms, illustrating how non-efflux antibacterial stress may generate misleading potentiation signals (Liu et al., 2023). Cumin and cinnamon preparations have been associated with *acrB*/*acrD* expression changes in Salmonella, but the active component is unresolved and membrane/PMF artefacts remain plausible (El-Demerdash et al., 2024). For this class, phenolic hydroxyls, terpene size, lipophilicity and formulation become useful optimisation variables only when the phenotype survives NPN/PI, membrane-potential, ATP, growth and viability controls and is reproduced in pump-deficient or pump-overexpressing strains. Delivery systems may improve exposure and solubility, but formulation cannot substitute for mechanism-specific validation.

### 4.4 Coumarins, quinones, phenolic acids and other scaffolds

Plumbagin and nordihydroguaiaretic acid (NDGA) show provisional AcrB-dependent evidence: in the primary study, antibiotic potentiation was observed in wild-type *Escherichia coli* but not in the Δ*acrB* strain, Nile Red efflux was inhibited, and the tested phytochemicals did not permeabilise the outer membrane (Ohene-Agyei et al., 2014). These findings satisfy the present Level 4a criterion for a pump-dependent phenotype, but the compounds do not reach Level 5 because their proposed hydrophobic-pocket poses have not been confirmed by direct binding, mutagenesis or structural analysis. Structural and dynamic studies of synthetic AcrB inhibitors provide a useful interpretive reference but do not independently validate the binding modes of plumbagin or NDGA (Reading et al., 2020; Tam et al., 2021). Camalexin provides another clear recent pump-dependent example: it increased ethidium accumulation without parallel membrane permeabilisation and lost potentiating activity in an AcrB-inactive background, supporting provisional Level 4a, although direct binding, mutagenesis and *in vivo* confirmation remain absent (Irianti et al., 2025). Chlorogenic acid potentiated levofloxacin against *K. pneumoniae in vitro* and in a mouse infection model (Tan et al., 2020), but no isogenic pump strains, accumulation assay or membrane/PMF controls isolated an RND mechanism. It therefore remains Level 2: animal efficacy alone is not Level 5 unless the outcome is demonstrably pump-linked. (+)-Usnic acid suppresses *adeB* expression and potentiates tigecycline in *A. baumannii*, but this is expression-level association (Level 4b), not proof of direct AdeB inhibition (Bankan et al., 2021). Quinones, coumarins and phenolic acids also require redox, Michael-acceptor and membrane controls, together with direct binding and pocket-mutant testing.

### 4.5 Semisynthetic natural product derivatives

Semisynthesis can bridge a weak or promiscuous natural-product hit to a more selective EPI-like lead. Quercetin derivatives, amino-chalcones and ursolic-acid derivatives show that polarity, lipophilicity, hydrogen-bonding capacity, charge, metabolic stability and toxicity can be tuned (Dwivedi et al., 2015; Lee et al., 2024; Souza et al., 2024). To avoid repeating class-specific wish lists, the common matched-pair design and validation priorities are consolidated in Section 6.1 and Table 5. The essential principle is to change one mechanism-relevant feature at a time while holding the biological system constant; otherwise apparent structure-feature trends remain descriptive rather than causal.

**Table 5:** Mechanism-informed optimisation considerations for major natural product-derived EPI-like classes.

Candidate class	Representative examples	Main evidence signal	Major confounder	Structure-feature considerations	Conservative interpretation	Next validation priority
Flavonoids / polyphenols	Quercetin, EGCG, chalcones	Antibiotic potentiation; occasional dye accumulation or mixed efflux/carbapenemase phenotypes	Membrane/PMF effects; polyphenol promiscuity; metal chelation; incomplete pump controls	Planar aromatic A–C system; C2 = C3 double bond; 4-keto group; OH/OMe pattern; aglycone vs. glycoside; LogD/PSA balance	Optimisation starting points; not sufficiently validated pump-associated RND EPI-like candidates	Matched analogue pairs; pump-mutant panels; membrane/PMF controls; LC–MS/MS accumulation; direct target assays
Alkaloids	Berberine, di-berberine probes, solasodine, lysergol	Potentiation, accumulation or probe evidence; di-berberine supports MexXY-OprM-associated aminoglycoside potentiation	Cytotoxicity; membrane activity; substrate-like transport risk; NorA bias; therapeutic-window uncertainty	Cationic/basic N; aromatic planarity; possible acidic-residue/pocket interactions; linked cationic units as probes	Mechanistically plausible but often probe-like; adjuvant claims require selectivity and safety	Separate inhibition from substrate behaviour; toxicity/PK; pump dependence; direct binding; in vivo combination testing
Terpenoids / essential-oil components	Carvacrol, thymol, nerolidol, farnesol, bisabolol	Efflux-like potentiation; expression changes; formulation-enhanced exposure	Membrane permeabilisation; PMF dissipation; volatility; active-principle ambiguity; formulation may mask toxicity	High lipophilicity; phenolic OH; terpene size; formulation dependence; membrane-liability risk	Useful adjuvant/formulation leads, but not direct RND evidence without strict controls	NPN/PI, membrane-potential, ATP, viability and isogenic pump controls; define active principles
Quinones / coumarins / phenolic acids	Plumbagin, NDGA, chlorogenic acid	Provisional AcrB-dependent phenotypes for plumbagin/NDGA; infection-model signal for chlorogenic acid; docking-supported pocket rationale	Redox cycling; Michael-acceptor reactivity; oxidative stress; cytotoxicity; expression-only evidence	Aromatic/planar core; proposed AcrB-pocket engagement; redox-tunable scaffold features; hydroxy/carboxyl/methoxy patterns	Plumbagin/NDGA are pump-associated Level 4a candidates without direct target proof; chlorogenic acid remains mechanism-generating	Less reactive analogues; pocket mutants; binding/competition assays; membrane/PMF controls; in vivo mechanism linkage
Indole phytoalexins	Camalexin, brassinin	Camalexin shows reported AcrB-dependent phenotype with ethidium accumulation and no membrane permeabilisation in the primary report	Single-study dependence; limited analogue series; no direct binding, mutagenesis, PK/toxicity or in vivo data	Compact indole/heteroaromatic core; low membrane-liability signal but limited structure-feature testing	One of the clearest recent natural-product AcrB-associated examples, but provisional rather than settled	Independent replication; analogue series; direct binding and pocket mutants; clinical MDR isolates; PK/toxicity and infection models

The table summarises evidence signals, structure-feature hypotheses, major confounders, conservative interpretations and validation priorities. These associations are hypothesis-generating and should not be interpreted as validated pharmacophores or formal structure–activity relationships. EGCG, epigallocatechin gallate; LC–MS/MS, liquid chromatography–tandem mass spectrometry; LogD, pH-dependent distribution coefficient; MDR, multidrug resistant; NDGA, nordihydroguaiaretic acid; OMe, methoxy group; OH, hydroxy group; PK, pharmacokinetics; PMF, proton-motive force; PSA, polar surface area.

### 4.6 Cross-class evidence distribution and antibiotic potentiation spectrum

Across the classes reviewed, natural product-derived EPI-like candidates have been associated with potentiation of fluoroquinolones, tetracyclines, beta-lactams, aminoglycosides and tigecycline. The evidence distribution in Figure 3—L2 (*n* = 2), L2–3 (*n* = 3), L3 (*n* = 1), L4a (*n* = 3), L4b (*n* = 1) and L5 (*n* = 0)—shows that reported synergy substantially outnumbers pump-associated or direct evidence. The most interpretable pairings use a well-characterised RND substrate together with strains in which the relevant pump is deleted, overexpressed or complemented. Claims involving colistin require particular caution because outer-membrane disruption can dominate the phenotype (Sundaramoorthy et al., 2019). Tables 3, 4 therefore separate core or near-core evidence from auxiliary evidence used to generate structure–activity relationship (SAR) hypotheses, and they identify the control or validation step needed to advance each candidate.

## 5 Cross-cutting mechanisms, confounders and validation toolbox

The class-specific evidence is integrated in Section 4; this section addresses only the cross-cutting mechanisms and controls needed to distinguish them (Figure 4). Natural products may alter RND-associated resistance through pocket occupation, substrate competition, conformational locking, assembly disruption, PMF interference, expression change or membrane perturbation. These routes generate overlapping phenotypes and must not be treated as equivalent claims. Synthetic EPIs such as PAβN, pyranopyridines and pyridylpiperazines remain benchmarks for validation depth, but they do not prove that a natural product shares the same mechanism (Opperman and Nguyen, 2015).

**Figure 4:**
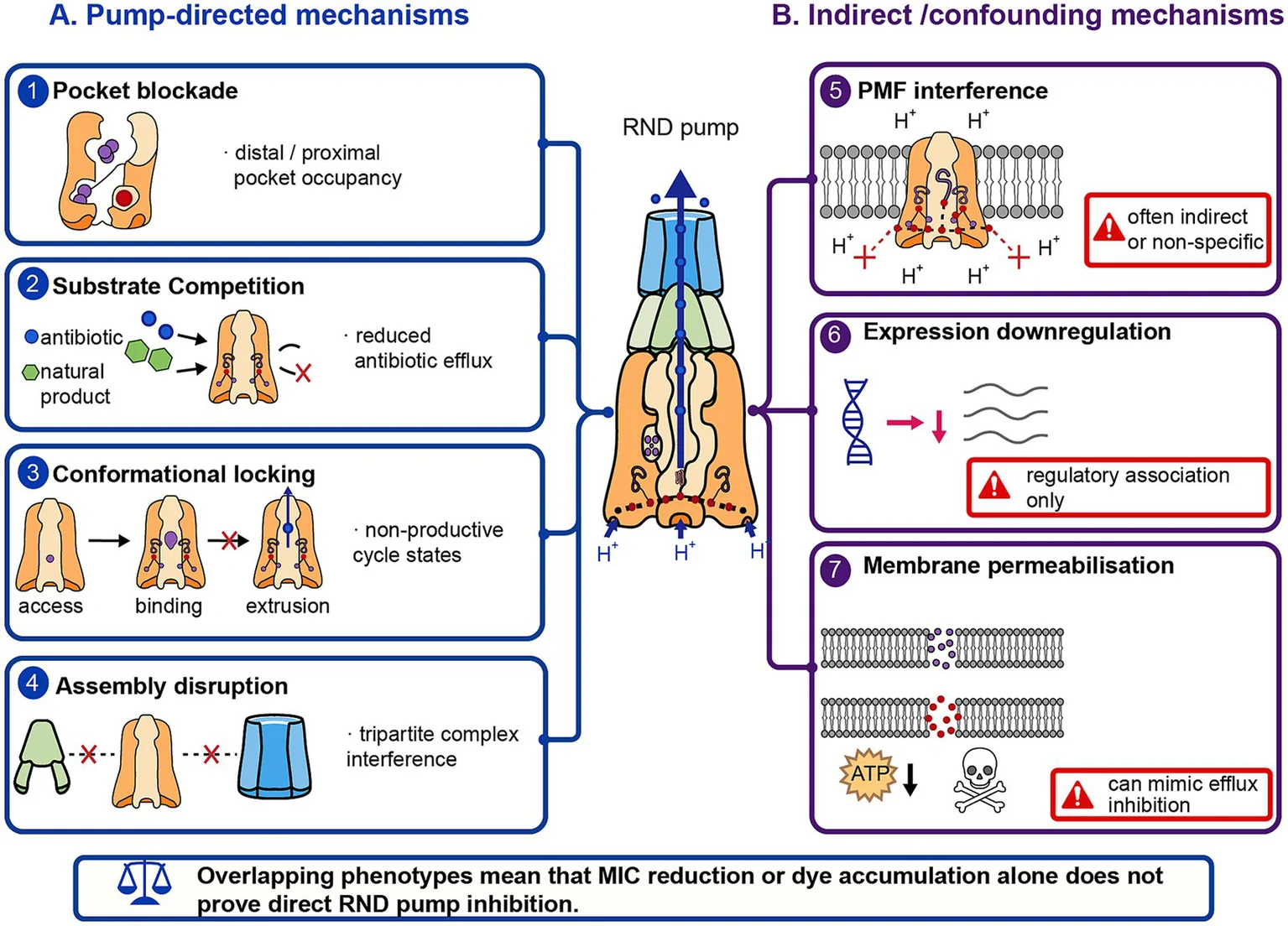
Simplified mechanisms of natural product-mediated modulation of RND-associated antibiotic resistance. (**A**) Pump-directed mechanisms include pocket blockade, substrate competition, conformational locking and assembly disruption. (**B**) Indirect or confounding mechanisms include PMF interference, expression-level regulation and membrane permeabilisation or stress-related effects. The figure emphasises that similar phenotypes can arise from distinct mechanisms and should not all be interpreted as direct RND inhibition.

### 5.1 Direct blockade of substrate-binding pockets

The best-characterised mechanism is direct occupation of a substrate-binding pocket. Among natural products the clearest pocket-directed cases are the AcrB-associated scaffolds of Section 4.4; for the rest, the binding claim rests on docking alone and stays at Level 1 until binding, mutagenesis or structure backs it up. What decides the matter is not synergy but a demonstration that the compound sits in a defined pocket—co-structures, residue-level mutagenesis or competition with a known pocket ligand. Structural studies of synthetic inhibitors show how such compounds settle into the AcrB distal pocket and hydrophobic trap (Nakashima et al., 2013; Vargiu et al., 2014; Sjuts et al., 2016). Structure-based virtual-screening campaigns against AcrB and AdeB have begun to nominate new pocket-directed chemotypes, though these still need the same biological confirmation asked of any natural-product candidate (Tuo et al., 2023).

### 5.2 Competitive interference with antibiotic extrusion

Some natural products may behave as pump substrates or competitive inhibitors, cutting extrusion by competing for binding pockets or transport channels. Proving real competition takes efflux kinetics, substrate-displacement assays and pump-mutant validation, not synergy alone (Vargiu and Nikaido, 2012; Vargiu et al., 2014). The classical MexAB-OprM-specific inhibitors first developed in *P. aeruginosa* remain a useful yardstick for what substrate-competitive, pump-specific inhibition should be able to show (Yoshida et al., 2007).

### 5.3 Allosteric locking of conformational cycling

Because RND transport depends on access–binding–extrusion cycling, an inhibitor might work by stabilising a non-productive conformation. Synthetic pyridylpiperazine inhibitors show that allosteric transmembrane pockets can be exploited and that *in vivo* efficacy may become reachable after physicochemical and PK optimisation (Plé et al., 2022; Vieira Da Cruz et al., 2024).

For natural products, the label allosteric inhibitor should be reserved for compounds with structural or mutational support. Piperazine arylideneimidazolones and pyrrole-based RND inhibitors provide examples of the evidence needed for a convincing allosteric or competitive claim (Bohnert et al., 2016; Mahey et al., 2024).

### 5.4 PMF / proton-relay interference

Natural products can also lower RND activity indirectly, by dissipating the PMF—but PMF effects are non-specific and may simply mean membrane toxicity. Membrane-potential, ATP, growth, viability and membrane-integrity assays are needed before potentiation can be put down to pump-specific interference with proton coupling (Opperman et al., 2014; Blair and Piddock, 2016; Plé et al., 2022).

### 5.5 Disruption of pump assembly

Breaking up the assembly of AcrA-AcrB-TolC, MexA-MexB-OprM or AdeA-AdeB-AdeC would be an appealing strategy, but the natural product evidence is thin, and this reads better as a design opportunity than a demonstrated mode of action. Before assembly disruption can be claimed, candidate scaffolds need assembly assays, cross-linking, native mass spectrometry, mutagenesis or structural methods (Daury et al., 2016; Du et al., 2018).

### 5.6 Downregulation of efflux pump expression

Another route is to reduce pump abundance through regulatory networks, without binding the transporter. El-Demerdash et al. reported downregulation of *acrB*, *mdtB*, *acrD*, *soxS* and *marA* in Salmonella exposed to cumin and cinnamon essential oils, and Pun et al. described phytochemical effects on *acrB* expression in Pectobacterium (Pun et al., 2023; El-Demerdash et al., 2024). Bankan et al. reported that (+)-usnic acid inhibits *adeB* overexpression and acts as a tigecycline adjuvant in tigecycline-resistant *A. baumannii* (Bankan et al., 2021). These results establish pump association only at the level of expression. For a defined single compound such as (+)-usnic acid that means Level 4b; for an undefined essential-oil mixture, as with the cumin and cinnamon preparations, the assigned grade is conservatively held at Level 3 (Table 3), because the expression change cannot be pinned to a defined compound. In neither case is the pump shown to be required for the phenotype, so these data should not be read as a pump-dependent phenotype (Level 4a) or as pump-targeted inhibition. Lower mechanistic directness need not mean lower therapeutic potential—regulatory downregulation of pump expression could be a legitimate adjuvant strategy—but it should be evaluated and reported as a distinct, expression-level mechanism, separate from transporter inhibition.

### 5.7 Distinguishing true efflux inhibition from membrane permeabilisation

A rise in dye accumulation admits several readings: genuine efflux inhibition, greater outer-membrane permeability, inner-membrane damage, PMF dissipation or cell death. Terpenoids, essential-oil components, quinones and some polyphenols are especially apt to produce membrane effects (Blair and Piddock, 2016; Dias et al., 2022). Telling these apart calls for a defined set of controls—NPN (*N*-phenyl-1-naphthylamine) uptake, propidium iodide staining, membrane-potential measurement, ATP quantification, growth controls, pump knockout/overexpression strains, and benchmark EPI and membrane-active comparators (Lomovskaya et al., 2001; Iyer et al., 2018).

### 5.8 Mechanistic evidence toolbox

Separating these mechanisms from one another, and from the confounders of Section 5.7, rests on a panel of orthogonal assays rather than any single readout. Functionally, dye-accumulation and real-time efflux kinetics—read together with NPN and propidium-iodide controls, membrane-potential and ATP measurements, and isogenic pump-deletion, overexpression or complementation strains— establish whether an effect is efflux-related and pump-dependent rather than a membrane or viability artefact (Lomovskaya et al., 2001; Blair and Piddock, 2016; Iyer et al., 2018). Moving from pump association to mechanism then takes direct target engagement: biophysical binding (surface plasmon resonance, isothermal titration calorimetry or microscale thermophoresis), residue-level mutagenesis of the distal pocket and hydrophobic trap, competition with a benchmark inhibitor and resistant-mutant selection test whether the compound acts at the proposed site, while cryo-EM or X-ray co-structures and molecular-dynamics simulation localise the interaction (Nakashima et al., 2013; Vargiu et al., 2014; Sjuts et al., 2016). Quantitative LC–MS/MS measurement of intracellular drug then confirms that any putative inhibition actually raises antibiotic accumulation (Geddes et al., 2021). Mapping a candidate onto this toolbox makes plain which evidence level (Table 2) its mechanistic claim can support, and which experiments would raise it.

## 6 Cross-class structure-feature opportunities and candidate prioritisation

### 6.1 Shared structure-feature gaps and opportunities

Across classes, the current literature supports qualitative, mechanism-informed structure-feature hypotheses rather than a validated quantitative SAR model. Recurring variables include aromatic or partly planar character, hydrogen-bonding capacity, cationic or ionisable groups, lipophilicity, polar surface area, solubility, three-dimensional shape and liabilities such as membrane activity, PMF dissipation, redox cycling or Michael-acceptor reactivity. These variables should be tested through matched analogue or formulation pairs in the same biological system, varying one feature at a time. A common readout panel should include sub-inhibitory potentiation, accumulation or efflux, membrane and PMF controls, pump-strain dependence, viability, cytotoxicity/haemolysis, solubility and—after prioritisation—direct target engagement. This consolidated design replaces repeated class-specific lists and provides a route from descriptive association to testable causality.

### 6.2 Gram-negative penetration and intracellular accumulation

Before a candidate can modulate an RND transporter it has to reach the periplasmic, membrane-proximal or transporter-associated site that matters for the phenotype. Outer-membrane influx is governed largely by porin size, charge and permeability rules, while RND pumps actively clear many amphipathic or hydrophobic molecules (Richter et al., 2017; Parker et al., 2019; Prajapati et al., 2021). Empirical accumulation guidelines for *E. coli* suggest molecules enter more readily when they carry an ionisable nitrogen, stay relatively rigid and keep limited three-dimensional globularity; these rules should be applied to natural products with care, since easier entry can also raise toxicity or membrane effects (Richter et al., 2017; Parker et al., 2019). Quantitative LC–MS/MS accumulation assays are especially informative, because they show whether an adjuvant truly raises intracellular antibiotic rather than just altering dye fluorescence, and optimisation should pair potentiation data with such measurements wherever it can (Iyer et al., 2018; Geddes et al., 2021). Much of this optimisation can proceed without drawn structures. The variables that count are reproducible sub-inhibitory potentiation, no compound-alone antibacterial artefact, negative membrane/PMF controls, increased intracellular antibiotic accumulation, pump-dependent loss or gain of effect, acceptable mammalian-cell toxicity, a haemolysis control, solubility, metabolic stability and compatibility with the intended antibiotic partner. The structure-related variables defined in Section 6.1 can still be described in text, but they are best used to steer validation rather than to assert a confirmed pharmacophore.

### 6.3 Testable structure-feature model and candidate prioritisation

Figure 5 summarises a testable, mechanism-informed structure-feature model without depicting chemical structures. Panel A links broad candidate-feature categories to plausible RND biology—pocket engagement, substrate-pathway competition, conformational effects, assembly or regulatory effects, and PMF/proton-relay interference. Panel B separates the evidence dimensions required for interpretation: efflux function, pump dependence, membrane/PMF controls, target engagement and translation. Panel C shows the practical progression from antibiotic potentiator to efflux-modulating, pump-associated and translational candidate.

**Figure 5:**
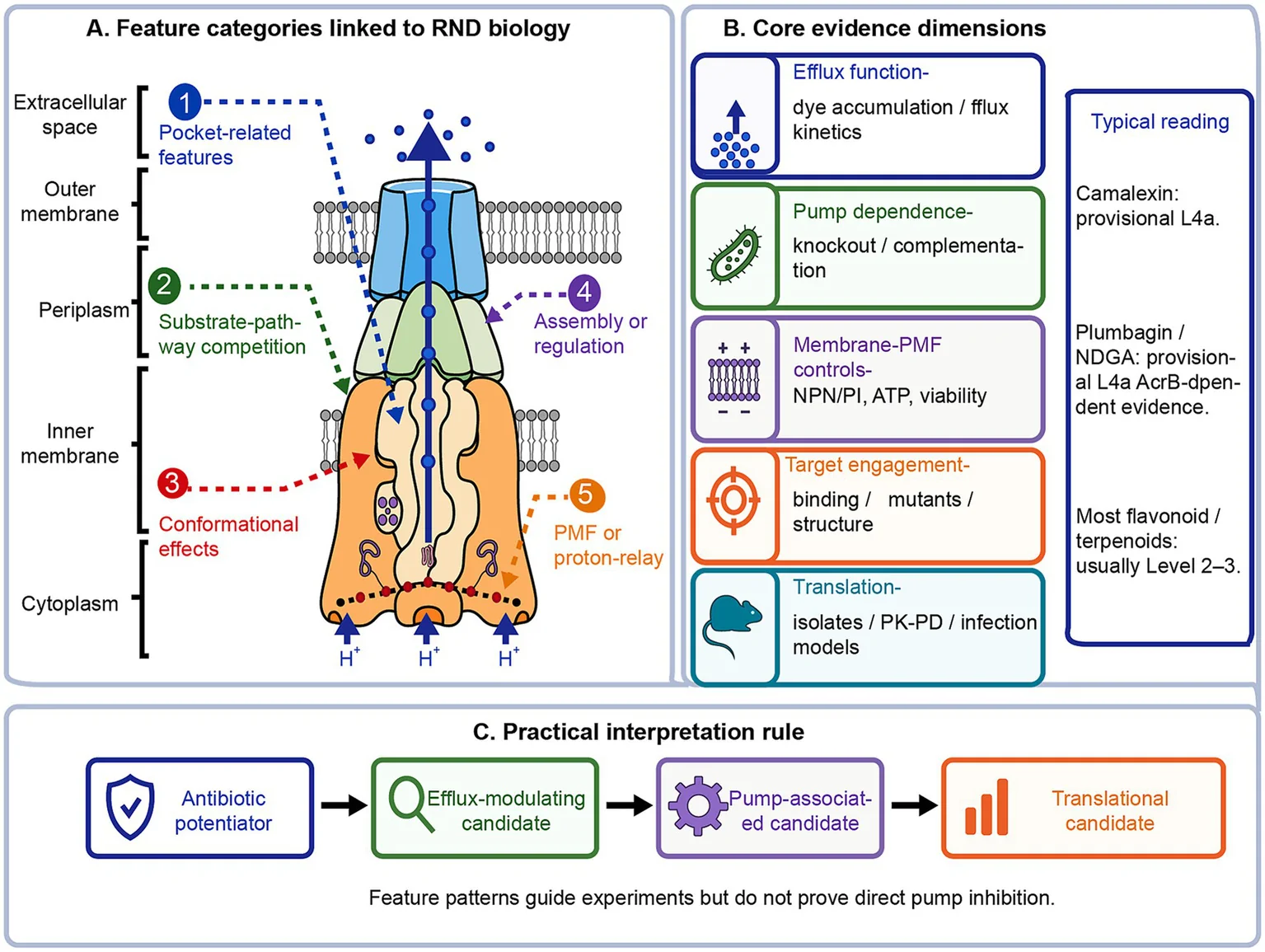
Simplified mechanism-informed structure-feature model for natural product-derived RND EPI-like candidates. **(A)** Broad candidate-feature categories are linked to plausible RND biology. **(B)** Evidence is assessed across efflux function, pump dependence, membrane/PMF controls, target engagement and translational relevance. **(C)** A practical pathway connects early antibiotic potentiators to efflux-modulating, pump-associated and translational candidates. Structure-feature associations are intended to guide validation and optimisation, not to establish direct target engagement on their own.

Detailed physicochemical variables are retained in Section 6.1 and Table 5 rather than crowded into the figure. The model is hypothesis-generating: it guides matched-pair experiments but does not establish direct binding, a pharmacophore or cross-pump transferability.

Candidate prioritisation should move beyond single-compound synergy (Table 5) and aim to improve water solubility, reduce membrane toxicity, strengthen pump-associated selectivity, optimise PK/PD, select the best antibiotic partner and establish a therapeutic window. Synthetic EPI programmes set useful benchmarks: pyranopyridine potency increased with improved AcrB pocket interactions, and optimisation of pyridylpiperazines enabled *in vivo* efficacy (Nguyen et al., 2015; Sjuts et al., 2016; Vieira Da Cruz et al., 2024). Natural-product programmes should apply the same discipline through semisynthesis, direct target assays, ADMET assessment and clinical MDR isolates. Co-development with a defined antibiotic partner may improve exposure planning and resistance management (Domalaon et al., 2018). Compounds dominated by redox or membrane activity should be deprioritised unless a selective pump-associated effect survives orthogonal controls.

## 7 Discussion: translational challenges and future perspectives

### 7.1 Why no bacterial EPI has reached clinical use

No bacterial EPI has reached approval (Table 6). The reasons are familiar: compounds active *in vitro* often fail *in vivo*; toxicity or membrane disruption caps dosing; PK/ADME is frequently poor; combination dosing is complex; indications and breakpoints are hard to define; and MDR isolates carry several overlapping resistance mechanisms at once (Sharma et al., 2019; Compagne et al., 2023; Duffey et al., 2024). Synthetic EPIs have sharpened the mechanistic picture without yet yielding an approved drug. The more encouraging signal is the recent generation of optimised allosteric inhibitors, which shows that *in vivo* efficacy becomes reachable once potency, selectivity and PK are tackled together (Vieira Da Cruz et al., 2024). *In vivo* proof of concept for adjuvant combinations is starting to appear in invertebrate and mammalian models—efflux-inhibitor/antibiotic combinations against MDR *P. aeruginosa* in *Galleria mellonella*, and ciprofloxacin–pentamidine against MDR Gram-negative bacteria—indicating that pump inhibition can translate into protection when exposure is adequate (Adamson et al., 2015; Fletcher et al., 2023).

**Table 6:** Translational barriers and proposed solutions for natural product-derived RND EPI-like candidates.

Barrier	Underlying cause	Consequence	Proposed solution	Priority
Poor in vivo efficacy	Low exposure, PK/ADME limits	*In vitro*–in vivo gap	PK optimisation, co-delivery	High
Membrane toxicity	Lipophilic, membrane-active scaffolds	Narrow therapeutic window	Reduce reactivity, formulate	High
Mechanistic ambiguity	MIC drop does not prove RND inhibition	Over-claimed pump-specific effects	Evidence grading, pump strains, direct target tests	High
Batch variability / solubility	Natural-source heterogeneity	Irreproducibility	Semisynthesis, standardisation	Medium
EPI resistance	Target mutation, regulatory shift	Loss of potentiation	Paired antibiotic–EPI design	Medium

Key barriers are linked to corresponding strategies for improving exposure, safety, mechanistic certainty, reproducibility and resistance management. ADME, absorption, distribution, metabolism and excretion; EPI, efflux pump inhibitor; MIC, minimum inhibitory concentration; PK, pharmacokinetics; RND, resistance-nodulation-division.

### 7.2 Natural product-specific liabilities

The translational liabilities are class-specific as well as general. Highly polar flavonoid glycosides may penetrate the Gram-negative envelope poorly; matched aglycones or semisynthetic analogues can improve lipophilicity, but they must be retested for membrane and PMF effects. Terpenoids and essential-oil components often have low aqueous solubility and membrane activity; liposomal or other nanodelivery systems can improve exposure, although free-compound and formulation controls are required so that delivery does not conceal intrinsic toxicity (Gupta et al., 2017; Oliveira-Tintino et al., 2023; Santana et al., 2023). Cationic berberine or di-berberine scaffolds may show substrate-like behaviour, non-selective accumulation or cytotoxicity; matched charge/linker analogues, pump-mutant selectivity, cytotoxicity/haemolysis and PK testing are needed before they can progress beyond chemical probes. Redox-active quinones such as plumbagin may combine apparent potentiation with oxidative or Michael-acceptor liabilities; less reactive analogues and parallel redox controls offer a direct way to determine whether pump-associated activity can be retained while toxicity is reduced. These examples provide a more precise context than a generic list of natural-product limitations. However, because well-validated examples of successful mitigation remain scarce in the natural-product RND EPI literature, these approaches should be viewed as experimentally testable mitigation strategies rather than established solutions.

### 7.3 Resistance evolution against EPIs

Bacteria can wear down EPI efficacy in several ways—target-site mutation, altered outer-membrane permeability, expression of alternative pumps, regulatory rewiring and metabolic adaptation (Colclough et al., 2020; Avakh et al., 2023; Dulanto Chiang and Dekker, 2024). Resistance-evolution experiments therefore belong early in optimisation, especially for compounds meant to be paired with a specific antibiotic. The inhibitor-resistance mutations seen in synthetic EPI studies are a useful warning for natural product programmes (Plé et al., 2022; Vieira Da Cruz et al., 2024). Stress responses can blunt adjuvant efficacy further; dying cells, for instance, can release signals that help surviving subpopulations weather antibiotic exposure, which again argues for running resistance- and tolerance-evolution experiments early (Bhattacharyya et al., 2020).

### 7.4 Future directions

Several complementary directions follow. Structure-guided reasoning should treat the AcrB/MexB/AdeB pockets, the hydrophobic trap and allosteric/transmembrane sites as hypothesis-generating templates, not as proof of direct natural-product binding. Computational approaches—AI, pharmacophore modelling and virtual screening of natural product libraries—need biological validation behind them and must not crowd out membrane/PMF controls or pump-specific strains. Semisynthesis should build matched analogue series rather than isolated derivatives. Nanodelivery and antibiotic-adjuvant co-delivery may help with solubility and exposure, but cannot be allowed to mask intrinsic toxicity. And *in vivo* validation with clinical MDR isolates, PK/PD analysis and resistance-evolution experiments should come before any claim of a translational lead. Physicochemical rules of Gram-negative permeation and siderophore-conjugate (Trojan-horse) delivery deserve to sit alongside efflux modulation, so that better pump engagement is matched by adequate intracellular accumulation (Loupias et al., 2023).

### 7.5 Practical decision gate for future natural product RND EPI studies

Before a hit is promoted, it should pass an explicit decision sequence (Figure 6): reproducible sub-inhibitory potentiation; exclusion of major membrane, PMF, growth and viability artefacts; demonstration of functional efflux modulation; and pump association or dependence in appropriate strains. A measured increase in intracellular antibiotic accumulation—preferably by LC–MS/MS where feasible—substantially strengthens advancement, but the method should be interpreted alongside the full control panel rather than treated as a stand-alone or universally mandatory assay.

**Figure 6:**
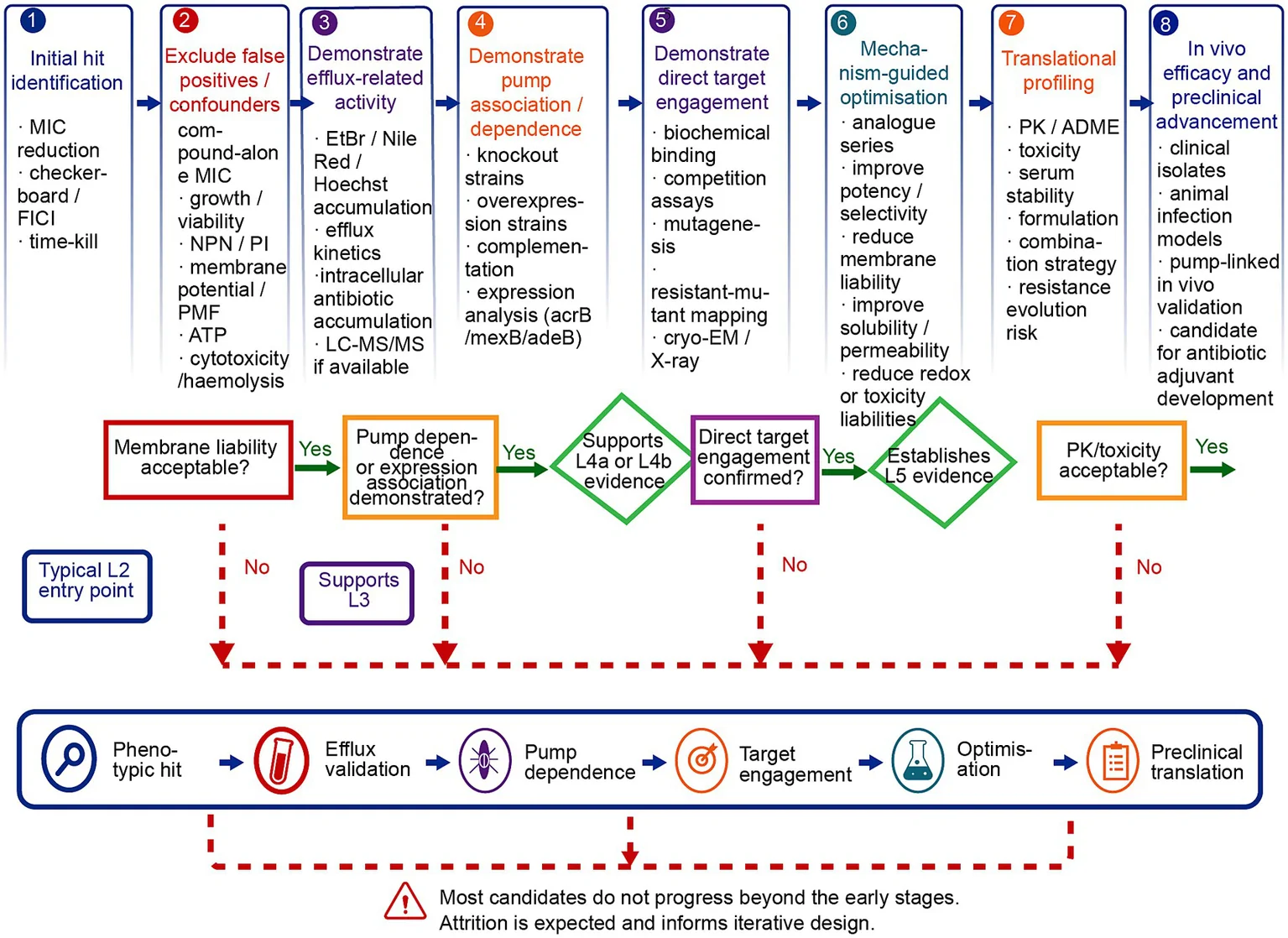
Staged validation roadmap for natural product-derived RND EPI-like antibiotic adjuvants. Initial screening is followed by exclusion of confounders, confirmation of efflux-related activity and pump-association testing. Direct target engagement supports Level 5 only when the proposed target mechanism is demonstrated. Quantitative LC–MS/MS measurement of intracellular antibiotic is strongly preferred where feasible, but it should be interpreted together with membrane, PMF, viability and pump-strain controls rather than treated as a universally mandatory stand-alone assay. PK/PD, resistance-selection studies and infection-model validation should follow only after mechanistic and preliminary safety criteria are met.

Candidates that clear the early gate then require direct target engagement, cytotoxicity/haemolysis, resistance-selection studies and testing in clinical MDR isolates. Animal infection models, PK/PD optimisation and antibiotic-partner selection follow only after mechanistic and preliminary safety criteria are met. This staged order is especially important for natural products, many of which are multi-target, redox-active or membrane-active and may show *in vitro* synergy without selective pump inhibition.

### 7.6 Limitations of this review

Several limitations should be kept in view. First, this is a narrative, evidence-graded synthesis rather than a systematic review. The search covered PubMed/MEDLINE, Web of Science Core Collection and Scopus, and English-language peer-reviewed work available up to 20 April 2026; non-English and grey-literature reports may therefore have been missed. Second, two authors graded candidates independently and resolved disagreements by discussion, but no kappa or other agreement statistic was calculated. The framework is consequently an explicit and auditable qualitative decision aid, not a quantitatively validated consensus instrument. Third, positive synergy studies are more likely to be published than negative results, so the apparent promise of natural-product efflux modulators may be overstated. Fourth, robust pump-associated data cluster heavily on *E. coli* AcrB. The structure-feature model and validation roadmap are therefore AcrB-anchored hypotheses, not validated general rules for MexB, MexY or AdeB. Extension to these clinically important pumps requires pump-matched knockout, overexpression or complementation strains; accumulation or efflux assays with membrane-integrity and PMF controls; and direct target-engagement or structural datasets for each pump. Fifth, cross-study heterogeneity in bacterial strains, antibiotic partners, compound purity, assay conditions, benchmark inhibitors and control panels limits numerical comparison. Finally, the structure-feature framework organises optimisation hypotheses but does not establish a formal pharmacophore or validated SAR model.

## 8 Conclusion

Natural product-derived RND EPI-like candidates remain a chemically diverse but undervalidated resource for antibiotic-adjuvant discovery against MDR Gram-negative bacteria. Potentiation reports are abundant, whereas few candidates have been examined with the controls needed to assign a pump-specific mechanism. The strongest Gram-negative RND-associated evidence still clusters on *E. coli* AcrB: camalexin is a clear but provisional pump-dependent example, while plumbagin and NDGA also show provisional Level 4a pump-dependent phenotypes but still lack direct target engagement; all remain AcrB-associated hypotheses. Evidence for MexB, MexY and AdeB is weaker and more fragmentary. Accordingly, the roadmap proposed here should be applied first as an AcrB-derived hypothesis and independently validated for each additional pump, rather than assumed to transfer across RND homologues. A fall in MIC should not be equated with RND inhibition because membrane permeabilisation, PMF dissipation, growth inhibition, efflux-gene downregulation and general stress can produce similar phenotypes.

A candidate should advance only after sub-inhibitory potentiation, exclusion of membrane and PMF artefacts, a demonstrated rise in intracellular antibiotic accumulation or fall in efflux, and a pump-dependent phenotype in isogenic or complemented strains. A Level 5 claim requires direct target engagement, mutational or structural validation, resistant-mutant analysis, or explicitly pump-linked *in vivo* efficacy. Toxicity, pharmacokinetic and resistance-selection assessments are additional requirements for translational advancement rather than components of the evidence grade itself. Progress will therefore depend on defined compounds, orthogonal controls, pump-specific strains, matched analogue or formulation series, direct target assays, toxicity and PK assessment, clinical MDR isolates, resistance-evolution experiments and infection models. Natural products remain useful starting points, but only candidates that climb this evidence hierarchy can credibly become antibiotic-adjuvant leads.

## References

[ref1] Adamson D. H. Krikstopaityte V. Coote P. J. (2015). Enhanced efficacy of putative efflux pump inhibitor/antibiotic combination treatments versus MDR strains of *Pseudomonas aeruginosa* in a *galleria mellonella* in vivo infection model. J. Antimicrob. Chemother. 70, 2271–2278. doi: 10.1093/jac/dkv111, 25921513

[ref2] Alav I. Kobylka J. Kuth M. S. Pos K. M. Picard M. Blair J. M. A. . (2021). Structure, assembly, and function of tripartite efflux and type 1 secretion Systems in Gram-Negative Bacteria. Chem. Rev. 121, 5479–5596. doi: 10.1021/acs.chemrev.1c00055, 33909410 PMC8277102

[ref3] Almatar M. Albarri O. Makky E. A. Köksal F. (2021). Efflux pump inhibitors: new updates. Pharmacol. Rep. 73, 1–16. doi: 10.1007/s43440-020-00160-9, 32946075

[ref4] Annunziato G. (2019). Strategies to overcome antimicrobial resistance (AMR) making use of non-essential target inhibitors: a review. Int. J. Mol. Sci. 20:5844. doi: 10.3390/ijms20235844, 31766441 PMC6928725

[ref5] Aron Z. Opperman T. J. (2018). The hydrophobic trap—the Achilles heel of RND efflux pumps. Res. Microbiol. 169, 393–400. doi: 10.1016/j.resmic.2017.11.001, 29146106 PMC5949246

[ref6] Athar M. Gervasoni S. Catte A. Basciu A. Malloci G. Ruggerone P. . (2023). Tripartite efflux pumps of the RND superfamily: what did we learn from computational studies? Microbiology 169:001307. doi: 10.1099/mic.0.001307, 36972322 PMC10191380

[ref7] Atzori A. Malloci G. Cardamone F. Bosin A. Vargiu A. V. Ruggerone P. (2020). Molecular interactions of Carbapenem antibiotics with the multidrug efflux transporter AcrB of *Escherichia coli*. Int. J. Mol. Sci. 21:860. doi: 10.3390/ijms21030860, 32013182 PMC7037162

[ref8] Avakh A. Grant G. D. Cheesman M. J. Kalkundri T. Hall S. (2023). The art of war with *Pseudomonas aeruginosa*: targeting Mex efflux pumps directly to strategically enhance antipseudomonal drug efficacy. Antibiotics 12:1304. doi: 10.3390/antibiotics12081304, 37627724 PMC10451789

[ref9] Bag A. Chattopadhyay R. R. (2014). Efflux-pump inhibitory activity of a gallotannin from *Terminalia chebula* fruit against multidrug-resistant uropathogenic *Escherichia coli*. Nat. Prod. Res. 28, 1280–1283. doi: 10.1080/14786419.2014.895729, 24620744

[ref10] Bankan N. Koka F. Vijayaraghavan R. Basireddy S. R. Jayaraman S. (2021). Overexpression of the *adeB* efflux pump gene in Tigecycline-resistant *Acinetobacter baumannii* clinical isolates and its inhibition by (+)Usnic acid as an adjuvant. Antibiotics (Basel) 10:1037. doi: 10.3390/antibiotics10091037, 34572620 PMC8472003

[ref11] Betts J. W. Wareham D. W. (2014). In vitro activity of curcumin in combination with epigallocatechin gallate (EGCG) versus multidrug-resistant *Acinetobacter baumannii*. BMC Microbiol. 14:172. doi: 10.1186/1471-2180-14-172, 24969489 PMC4083870

[ref12] Bharatham N. Bhowmik P. Aoki M. Okada U. Sharma S. Yamashita E. . (2021). Structure and function relationship of OqxB efflux pump from *Klebsiella pneumoniae*. Nat. Commun. 12:5400. doi: 10.1038/s41467-021-25679-0, 34518546 PMC8437966

[ref13] Bhattacharyya S. Walker D. M. Harshey R. M. (2020). Dead cells release a ‘necrosignal’ that activates antibiotic survival pathways in bacterial swarms. Nat. Commun. 11:4157. doi: 10.1038/s41467-020-17709-0, 32814767 PMC7438516

[ref14] Blair J. M. A. Piddock L. J. V. (2016). How to measure export via bacterial multidrug resistance efflux pumps. MBio 7:e00840-16. doi: 10.1128/mBio.00840-16 PMC4958252 27381291

[ref15] Bohnert J. A. Schuster S. Kern W. V. Karcz T. Olejarz A. Kaczor A. . (2016). Novel Piperazine Arylideneimidazolones inhibit the AcrAB-TolC pump in *Escherichia coli* and simultaneously act as fluorescent membrane probes in a combined real-time influx and efflux assay. Antimicrob. Agents Chemother. 60, 1974–1983. doi: 10.1128/AAC.01995-15, 26824939 PMC4808168

[ref16] Cernicchi G. Felicetti T. Sabatini S. (2021). Microbial efflux pump inhibitors: a journey around Quinoline and indole derivatives. Molecules 26:6996. doi: 10.3390/molecules26226996, 34834098 PMC8618814

[ref17] Cheesman M. Ilanko A. Blonk B. Cock I. (2017). Developing new antimicrobial therapies: are synergistic combinations of plant extracts/compounds with conventional antibiotics the solution? Pharmacogn. Rev. 11, 57–72. doi: 10.4103/phrev.phrev_21_17, 28989242 PMC5628525

[ref18] Cheng G. Dai M. Ahmed S. Hao H. Wang X. Yuan Z. (2016). Antimicrobial drugs in fighting against antimicrobial resistance. Front. Microbiol. 7:470. doi: 10.3389/fmicb.2016.00470, 27092125 PMC4824775

[ref19] Colclough A. L. Alav I. Whittle E. E. Pugh H. L. Darby E. M. Legood S. W. . (2020). RND efflux pumps in gram-negative Bacteria; regulation, structure and role in antibiotic resistance. Future Microbiol. 15, 143–157. doi: 10.2217/fmb-2019-0235, 32073314

[ref20] Compagne N. Vieira Da Cruz A. Müller R. T. Hartkoorn R. C. Flipo M. Pos K. M. (2023). Update on the discovery of efflux pump inhibitors against critical priority gram-negative Bacteria. Antibiotics 12:180. doi: 10.3390/antibiotics12010180, 36671381 PMC9854755

[ref21] Da Silva A. R. P. Costa M. D. S. Araújo N. J. S. De Freitas T. S. De Almeida R. S. Barbosa Filho J. M. . (2022). Potentiation of antibiotic action and efflux pump inhibitory effect on *Staphylococcus aureus* strains by solasodine. Antibiotics 11:1309. doi: 10.3390/antibiotics11101309, 36289967 PMC9598803

[ref22] Darby E. M. Trampari E. Siasat P. Gaya M. S. Alav I. Webber M. A. . (2023). Molecular mechanisms of antibiotic resistance revisited. Nat. Rev. Microbiol. 21, 280–295. doi: 10.1038/s41579-022-00820-y, 36411397

[ref23] Daury L. Orange F. Taveau J.-C. Verchère A. Monlezun L. Gounou C. . (2016). Tripartite assembly of RND multidrug efflux pumps. Nat. Commun. 7:10731. doi: 10.1038/ncomms10731, 26867482 PMC4754349

[ref24] Dias K. J. S. D. O. Miranda G. M. Bessa J. R. Araújo A. C. J. D. Freitas P. R. Almeida R. S. D. . (2022). Terpenes as bacterial efflux pump inhibitors: a systematic review. Front. Pharmacol. 13:953982. doi: 10.3389/fphar.2022.953982, 36313340 PMC9606600

[ref25] Domalaon R. Idowu T. Zhanel G. G. Schweizer F. (2018). Antibiotic hybrids: the next generation of agents and adjuvants against gram-negative pathogens? Clin. Microbiol. Rev. 31, e00077–e00017. doi: 10.1128/CMR.00077-17, 29540434 PMC5967690

[ref26] Dos Santos Barbosa C. R. Scherf J. R. De Freitas T. S. De Menezes I. R. A. Pereira R. L. S. Dos Santos J. F. S. . (2021). Effect of Carvacrol and Thymol on NorA efflux pump inhibition in multidrug-resistant (MDR) *Staphylococcus aureus* strains. J. Bioenerg. Biomembr. 53, 489–498. doi: 10.1007/s10863-021-09906-3, 34159523

[ref27] Du D. Wang-Kan X. Neuberger A. Van Veen H. W. Pos K. M. Piddock L. J. V. . (2018). Multidrug efflux pumps: structure, function and regulation. Nat. Rev. Microbiol. 16, 523–539. doi: 10.1038/s41579-018-0048-6, 30002505

[ref28] Duffey M. Jumde R. P. Da Costa R. M. A. Ropponen H.-K. Blasco B. Piddock L. J. V. (2024). Extending the potency and lifespan of antibiotics: inhibitors of gram-negative bacterial efflux pumps. ACS Infect. Dis. 10, 1458–1482. doi: 10.1021/acsinfecdis.4c00091, 38661541 PMC11091901

[ref29] Dulanto Chiang A. Dekker J. P. (2024). Efflux pump-mediated resistance to new beta lactam antibiotics in multidrug-resistant gram-negative bacteria. Commun. Med. 4:170. doi: 10.1038/s43856-024-00591-y, 39210044 PMC11362173

[ref30] Dwivedi G. R. Maurya A. Yadav D. K. Khan F. Darokar M. P. Srivastava S. K. (2015). Drug resistance reversal potential of Ursolic acid derivatives against Nalidixic acid- and multidrug-resistant *Escherichia coli*. Chem. Biol. Drug Des. 86, 272–283. doi: 10.1111/cbdd.12491, 25476148

[ref31] Eicher T. Seeger M. A. Anselmi C. Zhou W. Brandstätter L. Verrey F. . (2014). Coupling of remote alternating-access transport mechanisms for protons and substrates in the multidrug efflux pump AcrB. eLife 3:e03145. doi: 10.7554/eLife.03145, 25248080 PMC4359379

[ref32] El-Demerdash A. S. Kamel S. A. Elariny E. Y. T. Henidi H. Mahran Y. Alahdal H. . (2024). Natural inhibitors of Salmonella MDR efflux pumps AcrAB and AcrD: an integrated in Silico, molecular, and in vitro investigation. Int. J. Mol. Sci. 25:12949. doi: 10.3390/ijms252312949, 39684663 PMC11641668

[ref33] Ferrari R. G. Galiana A. Cremades R. Rodríguez J. C. Magnani M. Tognim M. C. B. . (2013). Expression of the *marA*, *soxS*, *acrB* and *ramA* genes related to the AcrAB/TolC efflux pump in *Salmonella enterica* strains with and without quinolone resistance-determining regions gyrA gene mutations. Braz. J. Infect. Dis. 17, 125–130. doi: 10.1016/j.bjid.2012.09.011, 23453941 PMC9427363

[ref34] Fletcher M. Mccormack A. Parcell B. J. Coote P. J. (2023). Combination therapy with ciprofloxacin and Pentamidine against multidrug-resistant *Pseudomonas aeruginosa*: assessment of in vitro and in vivo efficacy and the role of resistance-nodulation-division (RND) efflux pumps. Antibiotics 12:1236. doi: 10.3390/antibiotics12081236, 37627656 PMC10451767

[ref35] Geddes E. J. Li Z. Hergenrother P. J. (2021). An LC-MS/MS assay and complementary web-based tool to quantify and predict compound accumulation in *E. coli*. Nat. Protoc. 16, 4833–4854. doi: 10.1038/s41596-021-00598-y, 34480129 PMC8715754

[ref36] Glavier M. Puvanendran D. Salvador D. Decossas M. Phan G. Garnier C. . (2020). Antibiotic export by MexB multidrug efflux transporter is allosterically controlled by a MexA-OprM chaperone-like complex. Nat. Commun. 11:4948. doi: 10.1038/s41467-020-18770-5, 33009415 PMC7532149

[ref37] Górniak I. Bartoszewski R. Króliczewski J. (2019). Comprehensive review of antimicrobial activities of plant flavonoids. Phytochem. Rev. 18, 241–272. doi: 10.1007/s11101-018-9591-z

[ref38] Gupta D. Singh A. Khan A. U. (2017). Nanoparticles as efflux pump and biofilm inhibitor to rejuvenate bactericidal effect of conventional antibiotics. Nanoscale Res. Lett. 12:454. doi: 10.1186/s11671-017-2222-6, 28709374 PMC5509568

[ref39] Irianti M. I. Malloci G. Ruggerone P. Lodinsky E. V. Vincken J. P. Pos K. M. . (2025). Indole phytochemical camalexin as a promising scaffold for AcrB efflux pump inhibitors against *Escherichia coli*. Biomed. Pharmacother. 182:117779. doi: 10.1016/j.biopha.2024.117779, 39731937

[ref40] Irianti M. I. Vincken J. P. Van Dinteren S. Ter Beest E. Pos K. M. Araya-Cloutier C. (2023). Prenylated isoflavonoids from Fabaceae against the NorA efflux pump in *Staphylococcus aureus*. Sci. Rep. 13:22548. doi: 10.1038/s41598-023-48992-8, 38110428 PMC10728173

[ref41] Iyer R. Ye Z. Ferrari A. Duncan L. Tanudra M. A. Tsao H. . (2018). Evaluating LC-MS/MS to measure accumulation of compounds within Bacteria. ACS Infect. Dis. 4, 1336–1345. doi: 10.1021/acsinfecdis.8b00083, 29961312

[ref42] Kanagaratnam R. Sheikh R. Alharbi F. Kwon D. H. (2017). An efflux pump (MexAB-OprM) of *Pseudomonas aeruginosa* is associated with antibacterial activity of Epigallocatechin-3-gallate (EGCG). Phytomedicine 36, 194–200. doi: 10.1016/j.phymed.2017.10.010, 29157815

[ref43] Kavanaugh L. G. Dey D. Shafer W. M. Conn G. L. (2024). Structural and functional diversity of resistance-nodulation-division (RND) efflux pump transporters with implications for antimicrobial resistance. Microbiol. Mol. Biol. Rev. 88, e0008923–e0008923. doi: 10.1128/mmbr.00089-23, 39235227 PMC11426026

[ref44] Kavanaugh L. G. Mahoney A. R. Dey D. Wuest W. M. Conn G. L. (2023). Di-berberine conjugates as chemical probes of *Pseudomonas aeruginosa* MexXY-OprM efflux function and inhibition. NPJ Antimicrob. Resist. 1:12. doi: 10.1038/s44259-023-00013-4, 39843773 PMC11721654

[ref45] Klenotic P. A. Yu E. W. (2024). Structural analysis of resistance-nodulation cell division transporters. Microbiol. Mol. Biol. Rev. 88:e0019823. doi: 10.1128/mmbr.00198-23, 38551344 PMC11332337

[ref46] Kobylka J. Kuth M. S. Müller R. T. Geertsma E. R. Pos K. M. (2020). AcrB: a mean, keen, drug efflux machine. Ann. N. Y. Acad. Sci. 1459, 38–68. doi: 10.1111/nyas.14239, 31588569

[ref47] Kvist M. Hancock V. Klemm P. (2008). Inactivation of efflux pumps abolishes bacterial biofilm formation. Appl. Environ. Microbiol. 74, 7376–7382. doi: 10.1128/AEM.01310-08, 18836028 PMC2592912

[ref48] Lamut A. Peterlin Mašič L. Kikelj D. Tomašič T. (2019). Efflux pump inhibitors of clinically relevant multidrug resistant bacteria. Med. Res. Rev. 39, 2460–2504. doi: 10.1002/med.21591, 31004360

[ref49] Laws M. Shaaban A. Rahman K. M. (2019). Antibiotic resistance breakers: current approaches and future directions. FEMS Microbiol. Rev. 43, 490–516. doi: 10.1093/femsre/fuz014, 31150547 PMC6736374

[ref50] Lazarova M. Eicher T. Börnsen C. Zeng H. Athar M. Okada U. . (2025). Conformational plasticity across phylogenetic clusters of RND multidrug efflux pumps and its impact on substrate specificity. Nat. Commun. 16:11649. doi: 10.1038/s41467-025-66751-3, 41298458 PMC12749989

[ref51] Lee T. Lee S. Kim M. K. Ahn J. H. Park J. S. Seo H. W. . (2024). 3-O-substituted quercetin: an antibiotic-potentiating agent against multidrug-resistant gram-negative Enterobacteriaceae through simultaneous inhibition of efflux pump and broad-spectrum Carbapenemases. ACS Infectious Diseases 10, 1624–1643. doi: 10.1021/acsinfecdis.3c00715, 38652574

[ref52] Li Y. Ge X. (2023). Role of Berberine as a potential efflux pump inhibitor against MdfA from *Escherichia coli*: in vitro and in Silico studies. Microbiol. Spectrum 11, e03324–e03322. doi: 10.1128/spectrum.03324-22, 36786641 PMC10100983

[ref53] Li Y. Wen H. Ge X. (2021). Hormesis effect of Berberine against *Klebsiella pneumoniae* is mediated by up-regulation of the efflux pump KmrA. J. Nat. Prod. 84, 2885–2892. doi: 10.1021/acs.jnatprod.1c00642, 34665637

[ref54] Liu W. Chen G. Dou K. Yi B. Wang D. Zhou Q. . (2023). Eugenol eliminates carbapenem-resistant *Klebsiella pneumoniae* via reactive oxygen species mechanism. Front. Microbiol. 14:1090787. doi: 10.3389/fmicb.2023.1090787, 36876091 PMC9978153

[ref55] Lomovskaya O. Warren M. S. Lee A. Galazzo J. Fronko R. Lee M. . (2001). Identification and characterization of inhibitors of multidrug resistance efflux pumps in *Pseudomonas aeruginosa*: novel agents for combination therapy. Antimicrob. Agents Chemother. 45, 105–116. doi: 10.1128/AAC.45.1.105-116.2001, 11120952 PMC90247

[ref56] Loupias P. Laumaillé P. Morandat S. Mondange L. Guillier S. El Kirat K. . (2023). Synthesis and study of new siderophore analog-ciprofloxacin conjugates with antibiotic activities against *Pseudomonas aeruginosa* and Burkholderia spp. Eur. J. Med. Chem. 245:114921. doi: 10.1016/j.ejmech.2022.114921, 36399876

[ref57] Mahey N. Tambat R. Kalia R. Ingavale R. Kodesia A. Chandal N. . (2024). Pyrrole-based inhibitors of RND-type efflux pumps reverse antibiotic resistance and display anti-virulence potential. PLoS Pathog. 20:e1012121. doi: 10.1371/journal.ppat.1012121, 38593161 PMC11003683

[ref58] Masi M. Réfregiers M. Pos K. M. Pagès J.-M. (2017). Mechanisms of envelope permeability and antibiotic influx and efflux in gram-negative bacteria. Nat. Microbiol. 2:17001. doi: 10.1038/nmicrobiol.2017.1, 28224989

[ref59] Maurya A. Dwivedi G. R. Darokar M. P. Srivastava S. K. (2013). Antibacterial and synergy of Clavine alkaloid lysergol and its derivatives against Nalidixic acid-resistant *Escherichia coli*. Chem. Biol. Drug Des. 81, 484–490. doi: 10.1111/cbdd.12103, 23290001

[ref60] Murakami S. Nakashima R. Yamashita E. Matsumoto T. Yamaguchi A. (2006). Crystal structures of a multidrug transporter reveal a functionally rotating mechanism. Nature 443, 173–179. doi: 10.1038/nature05076, 16915237

[ref61] Murray C. J. L. Ikuta K. S. Sharara F. Swetschinski L. Robles Aguilar G. Gray A. . (2022). Global burden of bacterial antimicrobial resistance in 2019: a systematic analysis. Lancet 399, 629–655. doi: 10.1016/S0140-6736(21)02724-0 PMC8841637 35065702

[ref62] Naghavi M. Vollset S. E. Ikuta K. S. Swetschinski L. R. Gray A. P. Wool E. E. . (2024). Global burden of bacterial antimicrobial resistance 1990-2021: a systematic analysis with forecasts to 2050. Lancet 404, 1199–1226. doi: 10.1016/S0140-6736(24)01867-1 PMC11718157 39299261

[ref63] Nakashima R. Sakurai K. Yamasaki S. Hayashi K. Nagata C. Hoshino K. . (2013). Structural basis for the inhibition of bacterial multidrug exporters. Nature 500, 102–106. doi: 10.1038/nature12300, 23812586

[ref64] Nguyen S. T. Kwasny S. M. Ding X. Cardinale S. C. Mccarthy C. T. Kim H.-S. . (2015). Structure-activity relationships of a novel pyranopyridine series of gram-negative bacterial efflux pump inhibitors. Bioorg. Med. Chem. 23, 2024–2034. doi: 10.1016/j.bmc.2015.03.016, 25818767 PMC4398657

[ref65] Nishino K. Yamasaki S. Nakashima R. Zwama M. Hayashi-Nishino M. (2021). Function and inhibitory mechanisms of multidrug efflux pumps. Front. Microbiol. 12:737288. doi: 10.3389/fmicb.2021.737288, 34925258 PMC8678522

[ref66] Ohene-Agyei T. Mowla R. Rahman T. Venter H. (2014). Phytochemicals increase the antibacterial activity of antibiotics by acting on a drug efflux pump. MicrobiologyOpen 3, 885–896. doi: 10.1002/mbo3.212, 25224951 PMC4263512

[ref67] Oliveira-Tintino C. D. D. M. Santana J. E. G. Alencar G. G. Siqueira G. M. Gonçalves S. A. Tintino S. R. . (2023). Valencene, nootkatone and their liposomal nanoformulations as potential inhibitors of NorA, Tet(K), MsrA, and MepA efflux pumps in *Staphylococcus aureus* strains. Pharmaceutics 15:2400. doi: 10.3390/pharmaceutics15102400, 37896161 PMC10609713

[ref68] Opperman T. J. Kwasny S. M. Kim H.-S. Nguyen S. T. Houseweart C. D'souza S. . (2014). Characterization of a novel Pyranopyridine inhibitor of the AcrAB efflux pump of *Escherichia coli*. Antimicrob. Agents Chemother. 58, 722–733. doi: 10.1128/AAC.01866-13, 24247144 PMC3910843

[ref69] Opperman T. J. Nguyen S. T. (2015). Recent advances toward a molecular mechanism of efflux pump inhibition. Front. Microbiol. 6:421. doi: 10.3389/fmicb.2015.00421, 25999939 PMC4419859

[ref70] Ornik-Cha A. Wilhelm J. Kobylka J. Sjuts H. Vargiu A. V. Malloci G. . (2021). Structural and functional analysis of the promiscuous AcrB and AdeB efflux pumps suggests different drug binding mechanisms. Nat. Commun. 12:6919. doi: 10.1038/s41467-021-27146-2, 34824229 PMC8617272

[ref71] Pal A. Tripathi A. (2020). Quercetin inhibits carbapenemase and efflux pump activities among carbapenem-resistant gram-negative bacteria. APMIS 128, 251–259. doi: 10.1111/apm.13015 31755586

[ref72] Parker E. N. Drown B. S. Geddes E. J. Lee H. Y. Ismail N. Lau G. W. . (2019). Implementation of permeation rules leads to a FabI inhibitor with activity against gram-negative pathogens. Nat. Microbiol. 5, 67–75. doi: 10.1038/s41564-019-0604-5, 31740764 PMC6953607

[ref73] Plé C. Tam H.-K. Vieira Da Cruz A. Compagne N. Jiménez-Castellanos J.-C. Müller R. T. . (2022). Pyridylpiperazine-based allosteric inhibitors of RND-type multidrug efflux pumps. Nat. Commun. 13:115. doi: 10.1038/s41467-021-27726-2, 35013254 PMC8749003

[ref74] Prajapati J. D. Kleinekathöfer U. Winterhalter M. (2021). How to enter a bacterium: bacterial Porins and the permeation of antibiotics. Chem. Rev. 121, 5158–5192. doi: 10.1021/acs.chemrev.0c01213, 33724823

[ref75] Pun M. Khazanov N. Galsurker O. Kerem Z. Senderowitz H. Yedidia I. (2023). Inhibition of AcrAB-TolC enhances antimicrobial activity of phytochemicals in *Pectobacterium brasiliense*. Front. Plant Sci. 14:1161702. doi: 10.3389/fpls.2023.1161702, 37229130 PMC10203483

[ref76] Reading E. Ahdash Z. Fais C. Ricci V. Wang-Kan X. Grimsey E. . (2020). Perturbed structural dynamics underlie inhibition and altered efflux of the multidrug resistance pump AcrB. Nat. Commun. 11:5565. doi: 10.1038/s41467-020-19397-2, 33149158 PMC7642415

[ref77] Richter M. F. Drown B. S. Riley A. P. Garcia A. Shirai T. Svec R. L. . (2017). Predictive compound accumulation rules yield a broad-spectrum antibiotic. Nature 545, 299–304. doi: 10.1038/nature22308, 28489819 PMC5737020

[ref78] Salam M. A. Al-Amin M. Y. Salam M. T. Pawar J. S. Akhter N. Rabaan A. A. . (2023). Antimicrobial resistance: a growing serious threat for global public health. Healthcare 11:1946. doi: 10.3390/healthcare11131946, 37444780 PMC10340576

[ref79] Santana J. E. G. Oliveira-Tintino C. D. D. M. Gonçalves Alencar G. Siqueira G. M. Sampaio Alves D. Moura T. F. . (2023). Comparative antibacterial and efflux pump inhibitory activity of isolated nerolidol, farnesol, and α-bisabolol sesquiterpenes and their liposomal nanoformulations. Molecules 28:7649. doi: 10.3390/molecules28227649, 38005371 PMC10675182

[ref80] Sati H. Carrara E. Savoldi A. Hansen P. Garlasco J. Campagnaro E. . (2025). The WHO bacterial priority pathogens list 2024: a prioritisation study to guide research, development, and public health strategies against antimicrobial resistance. Lancet Infect. Dis. 25, 1033–1043. doi: 10.1016/S1473-3099(25)00118-5, 40245910 PMC12367593

[ref81] Seukep A. J. Kuete V. Nahar L. Sarker S. D. Guo M. (2020). Plant-derived secondary metabolites as the main source of efflux pump inhibitors and methods for identification. J. Pharm. Anal. 10, 277–290. doi: 10.1016/j.jpha.2019.11.002, 32923005 PMC7474127

[ref82] Seukep A. J. Mbuntcha H. G. Kuete V. Chu Y. Fan E. Guo M.-Q. (2022). What approaches to thwart bacterial efflux pumps-mediated resistance? Antibiotics 11:1287. doi: 10.3390/antibiotics11101287, 36289945 PMC9598416

[ref83] Shamsudin N. F. Ahmed Q. U. Mahmood S. Ali Shah S. A. Khatib A. Mukhtar S. . (2022). Antibacterial effects of flavonoids and their structure-activity relationship study: a comparative interpretation. Molecules 27:1149. doi: 10.3390/molecules27041149, 35208939 PMC8879123

[ref84] Sharma A. Gupta V. K. Pathania R. (2019). Efflux pump inhibitors for bacterial pathogens: from bench to bedside. Indian J. Med. Res. 149, 129–145. doi: 10.4103/ijmr.IJMR_2079_17, 31219077 PMC6563736

[ref85] Shi X. Chen M. Yu Z. Bell J. M. Wang H. Forrester I. . (2019). In situ structure and assembly of the multidrug efflux pump AcrAB-TolC. Nat. Commun. 10:2635. doi: 10.1038/s41467-019-10512-6, 31201302 PMC6570770

[ref86] Sjuts H. Vargiu A. V. Kwasny S. M. Nguyen S. T. Kim H.-S. Ding X. . (2016). Molecular basis for inhibition of AcrB multidrug efflux pump by novel and powerful pyranopyridine derivatives. Proc. Natl. Acad. Sci. 113, 3509–3514. doi: 10.1073/pnas.1602472113, 26976576 PMC4822567

[ref87] Souza M. A. D. Rodrigues L. G. Rocha J. E. De Freitas T. S. Bandeira P. N. Marinho M. M. . (2024). Synthesis, structural characterization, antibacterial and antibiotic-modifying activity, ADMET study, molecular docking and dynamics of chalcone (E)-1-(4-aminophenyl)-3-(4-nitrophenyl)prop-2-en-1-one in strains of *Staphylococcus aureus* carrying NorA and MepA efflux pumps. J. Biomol. Struct. Dyn. 42, 1670–1691. doi: 10.1080/07391102.2023.2213777 37222682

[ref88] Spengler G. Kincses A. Gajdács M. Amaral L. (2017). New roads leading to old destinations: efflux pumps as targets to reverse multidrug resistance in Bacteria. Molecules 22:468. doi: 10.3390/molecules22030468, 28294992 PMC6155429

[ref89] Stavri M. Piddock L. J. V. Gibbons S. (2007). Bacterial efflux pump inhibitors from natural sources. J. Antimicrob. Chemother. 59, 1247–1260. doi: 10.1093/jac/dkl460, 17145734

[ref90] Sundaramoorthy N. S. Suresh P. Selva Ganesan S. Ganeshprasad A. Nagarajan S. (2019). Restoring colistin sensitivity in colistin-resistant *E. coli*: combinatorial use of MarR inhibitor with efflux pump inhibitor. Sci. Rep. 9:19845. doi: 10.1038/s41598-019-56325-x, 31882661 PMC6934491

[ref91] Tacconelli E. Carrara E. Savoldi A. Harbarth S. Mendelson M. Monnet D. L. . (2018). Discovery, research, and development of new antibiotics: the WHO priority list of antibiotic-resistant bacteria and tuberculosis. Lancet Infect. Dis. 18, 318–327. doi: 10.1016/S1473-3099(17)30753-3, 29276051

[ref92] Tam H.-K. Foong W. E. Oswald C. Herrmann A. Zeng H. Pos K. M. (2021). Allosteric drug transport mechanism of multidrug transporter AcrB. Nat. Commun. 12:3889. doi: 10.1038/s41467-021-24151-3, 34188038 PMC8242077

[ref93] Tan S. Gao J. Li Q. Guo T. Dong X. Bai X. . (2020). Synergistic effect of chlorogenic acid and levofloxacin against Klebsiella pneumonia infection in vitro and in vivo. Sci. Rep. 10:20013. doi: 10.1038/s41598-020-76895-5, 33203903 PMC7672055

[ref94] Tiwari S. Jamal S. B. Hassan S. S. Carvalho P. V. S. D. Almeida S. Barh D. . (2017). Two-component signal transduction Systems of Pathogenic Bacteria as Targets for antimicrobial therapy: an overview. Front. Microbiol. 8:1878. doi: 10.3389/fmicb.2017.01878, 29067003 PMC5641358

[ref95] Tsutsumi K. Yonehara R. Ishizaka-Ikeda E. Miyazaki N. Maeda S. Iwasaki K. . (2019). Structures of the wild-type MexAB-OprM tripartite pump reveal its complex formation and drug efflux mechanism. Nat. Commun. 10:1520. doi: 10.1038/s41467-019-09463-9, 30944318 PMC6447562

[ref96] Tuo Y. Tang Y. Yang R. Zhao X. Luo M. Zhou X. . (2023). Virtual screening and biological activity evaluation of novel efflux pump inhibitors targeting AdeB. Int. J. Biol. Macromol. 250:126109. doi: 10.1016/j.ijbiomac.2023.126109, 37544561

[ref97] Vargiu A. V. Nikaido H. (2012). Multidrug binding properties of the AcrB efflux pump characterized by molecular dynamics simulations. Proc. Natl. Acad. Sci. 109, 20637–20642. doi: 10.1073/pnas.1218348109, 23175790 PMC3528587

[ref98] Vargiu A. V. Ruggerone P. Opperman T. J. Nguyen S. T. Nikaido H. (2014). Molecular mechanism of MBX2319 inhibition of *Escherichia coli* AcrB multidrug efflux pump and comparison with other inhibitors. Antimicrob. Agents Chemother. 58, 6224–6234. doi: 10.1128/AAC.03283-14, 25114133 PMC4187987

[ref99] Vieira Da Cruz A. Jiménez-Castellanos J.-C. Börnsen C. Van Maele L. Compagne N. Pradel E. . (2024). Pyridylpiperazine efflux pump inhibitor boosts in vivo antibiotic efficacy against *K. pneumoniae*. EMBO Mol. Med. 16, 93–111. doi: 10.1038/s44321-023-00007-9, 38177534 PMC10897476

[ref100] Waditzer M. Bucar F. (2021). Flavonoids as inhibitors of bacterial efflux pumps. Molecules 26:6904. doi: 10.3390/molecules26226904, 34833994 PMC8625893

[ref101] Wang Z. Fan G. Hryc C. F. Blaza J. N. Serysheva I. I. Schmid M. F. . (2017). An allosteric transport mechanism for the AcrAB-TolC multidrug efflux pump. eLife 6:e24905. doi: 10.7554/eLife.24905, 28355133 PMC5404916

[ref102] Wang Y. Venter H. Ma S. (2016). Efflux pump inhibitors: a novel approach to combat efflux-mediated drug resistance in Bacteria. Curr. Drug Targets 17, 702–719. doi: 10.2174/1389450116666151001103948, 26424403

[ref103] Wright G. D. (2016). Antibiotic adjuvants: rescuing antibiotics from resistance. Trends Microbiol. 24, 862–871. doi: 10.1016/j.tim.2016.06.009, 27430191

[ref104] Yoshida K.-I. Nakayama K. Ohtsuka M. Kuru N. Yokomizo Y. Sakamoto A. . (2007). MexAB-OprM specific efflux pump inhibitors in *Pseudomonas aeruginosa*. Part 7: highly soluble and in vivo active quaternary ammonium analogue D13-9001, a potential preclinical candidate. Bioorg. Med. Chem. 15, 7087–7097. doi: 10.1016/j.bmc.2007.07.039, 17869116

[ref105] Zgurskaya H. I. Rybenkov V. V. Krishnamoorthy G. Leus I. V. (2018). Trans-envelope multidrug efflux pumps of gram-negative bacteria and their synergism with the outer membrane barrier. Res. Microbiol. 169, 351–356. doi: 10.1016/j.resmic.2018.02.002, 29454787 PMC6095828

[ref106] Zhang Z. Morgan C. E. Bonomo R. A. Yu E. W. (2023). Cryo-EM structures of the *Klebsiella pneumoniae* AcrB multidrug efflux pump. MBio 14:e00659-23. doi: 10.1128/mbio.00659-23 PMC10294659 37067435

[ref107] Zuo Z. Wang B. Weng J. Wang W. (2015). Stepwise substrate translocation mechanism revealed by free energy calculations of doxorubicin in the multidrug transporter AcrB. Sci. Rep. 5:13905. doi: 10.1038/srep13905, 26365278 PMC4595977

[ref108] Zwama M. Yamaguchi A. (2018). Molecular mechanisms of AcrB-mediated multidrug export. Res. Microbiol. 169, 372–383. doi: 10.1016/j.resmic.2018.05.005, 29807096

